# Prediction Models for Future High-Need High-Cost Healthcare Use: a Systematic Review

**DOI:** 10.1007/s11606-021-07333-z

**Published:** 2022-01-11

**Authors:** Ursula W. de Ruijter, Z. L. Rana Kaplan, Wichor M. Bramer, Frank Eijkenaar, Daan Nieboer, Agnes van der Heide, Hester F. Lingsma, Willem A. Bax

**Affiliations:** 1 Section of Medical Decision Making, Department of Public Health, Erasmus MC, University Medical Center, Dr. Molewaterplein 40, 3015 GD Rotterdam, The Netherlands; 2 Department of Internal Medicine, Northwest Clinics, Alkmaar, The Netherlands; 3 Medical Library, Erasmus MC, University Medical Center, Rotterdam, The Netherlands; 4 Erasmus School of Health Policy & Management, Erasmus University Rotterdam, Rotterdam, The Netherlands; 5 Section of Care at the End of Life, Department of Public Health, Erasmus MC, University Medical Center, Rotterdam, The Netherlands

**Keywords:** prognosis, meaningful use, managed care programmes, patient care management, health expenditures

## Abstract

**Background:**

In an effort to improve both quality of care and cost-effectiveness, various care-management programmes have been developed for high-need high-cost (HNHC) patients. Early identification of patients at risk of becoming HNHC (i.e. case finding) is crucial to a programme’s success. We aim to systematically identify prediction models predicting future HNHC healthcare use in adults, to describe their predictive performance and to assess their applicability.

**Methods:**

Ovid MEDLINE® All, EMBASE, CINAHL, Web of Science and Google Scholar were systematically searched from inception through January 31, 2021. Risk of bias and methodological quality assessment was performed through the Prediction model Risk Of Bias Assessment Tool (PROBAST).

**Results:**

Of 5890 studies, 60 studies met inclusion criteria. Within these studies, 313 unique models were presented using a median development cohort size of 20,248 patients (IQR 5601–174,242). Predictors were derived from a combination of data sources, most often claims data (*n* = 37; 62%) and patient survey data (*n* = 29; 48%). Most studies (*n* = 36; 60%) estimated patients’ risk to become part of some top percentage of the cost distribution (top-1–20%) within a mean time horizon of 16 months (range 12–60). Five studies (8%) predicted HNHC persistence over multiple years. Model validation was performed in 45 studies (76%). Model performance in terms of both calibration and discrimination was reported in 14 studies (23%). Overall risk of bias was rated as ‘high’ in 40 studies (67%), mostly due to a ‘high’ risk of bias in the subdomain ‘Analysis’ (*n* = 37; 62%).

**Discussion:**

This is the first systematic review (PROSPERO CRD42020164734) of non-proprietary prognostic models predicting HNHC healthcare use. Meta-analysis was not possible due to heterogeneity. Most identified models estimated a patient’s risk to incur high healthcare expenditure during the subsequent year. However, case-finding strategies for HNHC care-management programmes are best informed by a model predicting HNHC persistence. Therefore, future studies should not only focus on validating and extending existing models, but also concentrate on clinical usefulness.

**Supplementary Information:**

The online version contains supplementary material available at 10.1007/s11606-021-07333-z.

## INTRODUCTION

The distribution of healthcare spending is skewed: a majority of expenditure is incurred by a small proportion of patients.^1,2^ In part, this is due to appropriate use of resources, but unfortunately, many high-cost patients receive unnecessary or ineffective care.^3^ Therefore, patients who remain high cost over multiple years, often characterized by complex medical and social needs, have become a target for intervention.^4,5^Care-management programmes for this so-called high-need high-cost(HNHC) population aim to improve quality of care and enhance cost-effectiveness.^5–7^ Programmes often encompass structured clinical follow-up supervised by an interdisciplinary care team, whether or not in combination with self-management support, pharmaceutical care and patient and caregiver education.^7,8^ Programme results vary, but a positive impact has been shown on quality of care, healthcare use and subsequently cost.^9–11^

Selecting appropriate patients is essential for the success of such programmes.^6,8^ Yet, HNHC patients comprise a very heterogeneous population, hindering clear definition of who is HNHC (i.e. case definition) and strategies for patient selection (i.e. case finding). Case definition has been based on prior healthcare utilization (e.g. prior hospitalizations), healthcare expenditure (e.g. top cost decile in previous year) or clinical profile (e.g. comorbidity score).^3,13–16^ One method for case finding is through quantitative prediction models.^12–14^ Predicting future healthcare utilization and spending has been important in actuarial science for decades. Consequently, proprietary claims–based risk assessment tools have been developed.^15,16^ In recent years, the purpose of these tools has shifted from risk adjustment to case finding for care-management programmes in an attempt to improve quality of care.^15,16^ However, proprietary tools are often commercial and therefore not freely available to those developing a care-management programme.

This systematic review identified and assessed current studies on non-proprietary models predicting future HNHC healthcare use. We aimed to evaluate model performance, appraise risk of bias and assess applicability as part of a case-finding strategy for HNHC care-management programmes.

## METHODS

This review was reported according to PRISMA guidelines and prospectively registered^17,18^ (PROSPERO CRD42020164734). Five databases were searched until January 31, 2021, for development and validation studies of prognostic prediction models with an explicitly described outcome on HNHC healthcare use. Two reviewers (UR, RK) independently performed study selection. We excluded studies not reporting on original research such as reviews, meta-analyses or methodological studies. Further exclusion criteria were as follows: published in a language other than English, population including minors, population with a single diagnosis (i.e. one specific morbidity as a condition), no provision of a definition of high-need high-cost healthcare use or a definition without any measure of cost. The eMethods in the Supplement provides a full description of included databases, study selection, data extraction and data synthesis. Authors of 21 potentially eligible studies were contacted to collect additional data or to answer methodological questions (eTable 3 in the Supplement).^19^ In five cases, this led to exclusion from this review because they met the exclusion criteria.

Predictors were described based on the type of data source they were derived from (e.g. claims data, survey data) and categorized according to Andersen’s Behavioural Model of Healthcare Utilization.^20^ This model describes healthcare use as a function of ‘*Predisposing*’, ‘*Enabling*’ and ‘*Need*’ characteristics. First, ‘*Predisposing*’ characteristics predispose people to healthcare use while not directly linked to such use and are divided into three categories: demographic (e.g. age, sex), social structure (e.g. education, occupation) and beliefs (e.g. values concerning health and illness).^20^ Second, ‘*Enabling*’ characteristics facilitate or inhibit use of services and are divided into two categories: family (e.g. income, insurance) and community (e.g. ratio of health personnel to population, urban-rural character).^20^ Finally, ‘*Need*’ characteristics are an assessment of whether or not illness requires care by the patient or healthcare provider and is divided into the perceived level of illness and the (clinically) evaluated level of illness.^20^ The eMethods provide a detailed description of Andersen’s model, its three categories of predictors and their subcategories.

Model performance was evaluated using the reported measures of discrimination (i.e. ability to discriminate between those with and those without the outcome), calibration (i.e. the agreement between observed and predicted outcomes), classification (i.e. sensitivity and specificity) and clinical usefulness (i.e. the ability to make better decisions with a model than without).^21,22^ Applicability for clinical use was assessed for all validated models by plotting model performance, indicated by their discrimination (expressed as C-statistic), against the expected performance in new patients, indicated by risk of overfitting to the development data (expressed as natural log of the events per variable (EPV)). A C-statistic ≥ 0.7 implies good discrimination.^23–25^ An EPV ≥ 20 (natural log = 3) is considered to imply minimal risk of overfitting.^26,27^

Risk of bias and concerns regarding the applicability of a primary study to the review question were assessed independently by two reviewers (UR, RK) through Prediction model Risk Of Bias Assessment Tool (PROBAST).^28^ Outcomes were summarized as ‘*high*’, ‘*uncertain*’ and ‘*low*’ risk or concern.^28^

## RESULTS

Of 5890 unique studies reviewed, 530 were selected for full-text review and 60 met all criteria (eFigure 1). These 60 studies provide information on the development and evaluation of 313 unique models predicting future HNHC healthcare use (Table 1). Fifteen studies (25%) developed a model without further validation; 25 studies (42%) developed a model and conducted internal validation; 15 studies (25%) developed a model and conducted external validation and five studies (8%) validated an existing model. All were cohort studies, of which 20 were prospective (33%). In development cohorts, the population size ranged from 136 to 10,300,856. Most studies (*n* = 42; 70%) were performed in the USA, of which 11 studies (18%) were based on data from the Centers for Medicare and Medicaid Services (CMS): five on Medicare data, four on Medicaid data and two on a combination. Other studies originated from Canada (*n* = 8; 13%), Spain (*n* = 2; 3%), Denmark (*n* = 1; 2%), Japan (*n* = 1; 2%), the Netherlands (*n* = 1; 2%), Singapore (*n* = 1; 2%), South Korea (*n* = 1; 2%), Switzerland (*n* = 1; 2%), Taiwan (*n* = 1; 2%) and the UK (*n* = 1; 2%). Most studies used regression analysis (*n* = 47; 78%), but a substantial number of studies (also) employed artificial intelligence (*n* = 14; 23%). For each study, a full description of the study population, predicted outcome, sample size, predictors, prediction timespan and performance measures is available in eTable 1 in the Supplement.

**Table 1:** Summary of Development, Validation and Extension Studies of Identified Models Predicting High-Need High-Cost Healthcare Use

	Development only with or without internal validation (*n* = 40 studies)	Development and external validation (*n* = 15 studies)	External validation only (*n* = 5 studies)
Number of models, mean (range per study)	5 (1–21)	6 (1–33)	6 (1–20)
Prospective data, no. of studies (%)	14 (35%)	3 (20%)	3 (60%)
Study population			
Development cohort, median (IQR)	18,065 (3255–191,758)	36,316 (7948–104,500)	–
Validation cohort, median (IQR)	21,431 (3915–164,738)	14,798 (7237–122,727)	83,187 (10,504–11,684,427)
Population only > 65 years of age, no. of studies (%)	9 (23%)	2 (13%)	0 (0%)
Population setting			
General population, no. of studies (%)	16 (40%)	9 (60%)	4 (80%)
Medicare or Medicaid, no. of studies (%)	8 (20%)	3 (20%)	0 (0%)
Primary care, no. of studies (%)	8 (20%)	0 (0%)	1 (20%)
Other^*^, no. of studies (%)	8 (20%)	3 (20%)	0 (0%)
Source of predictor data^†^			
Insurance claims data, no. of studies (%)	24 (60%)	8 (53%)	4 (80%)
Survey data, no. of studies (%)	20 (50%)	8 (53%)	1 (20%)
Electronic health records, no. of studies (%)	12 (30%)	4 (27%)	4 (80%)
Other^‡^, no. of studies (%)	4 (10%)	3 (20%)	1 (20%)
Outcome			
Prediction timespan, mean (range), months	13 (1–60)	16 (6–60)	22 (12–60)
Prediction timespan beyond 12 months, no. of studies (%)	2 (5%)	2 (13%)	1 (20%)

*IQR* interquartile range, *HNHC*high-need high-cost healthcare use

*For example, hospital inpatient care, Veterans Affairs (VA) healthcare service, caring homes

†As some studies use predictors from more than one data source, totals may add up to > 100%

‡For example, death registries, Veterans Affairs (VA) healthcare service, clinical laboratory database, pharmacy claims data

### Risk Predictors

Predictors were generally derived from a combination of data sources, with claims data (*n* = 36; 60%) and survey data (*n* = 29; 48%) used most often. Classification of predictors according to Andersen’s model showed that all studies used ‘*Predisposing*’ predictors. In this category, demographics (e.g. age, sex) were most common while beliefs were included in none of the studies. ‘*Enabling*’ predictors (e.g. income, access to regular source) were used in 23 studies (38%), and ‘*Need*’ predictors were included in 53 studies (88%). Perceived need was predominantly represented in studies based on survey data. Finally, predictors based on prior cost or healthcare utilization were included in 42 studies (70%).

### Predicted Outcome and Associated Timespan

Most studies (*n* = 36; 60%) estimated the risk for patients to become part of some top percentage of the cost distribution (e.g. top decile) within a mean time horizon of 16 months (range 12–60). Other outcomes were measures of healthcare utilization (e.g. top decile of care visits) (*n* = 23; 38%) and alternative measures of expenditure (e.g. total healthcare cost) (*n* = 12; 20%). Most studies (*n* = 51; 85%) had a prediction timespan of 12 months: they made predictions at baseline for the next 12 months. Four studies (7%) had a shorter prediction timespan while five studies (8%) extended their timespan beyond 12 months with a focus on the prediction of persistence in HNHC healthcare use. Two of these five studies concerned the same model.^29,30^

### Model Performance

Model validation was provided in 45 studies (75%): 25 studies (42%) conducted internal and 20 studies (33%) conducted external validation. Internal validation was most often performed by means of a split-sample analysis (*n* = 17; 28%), in which a model’s predictive performance is evaluated on a random part of the study sample after being developed on the complimentary part (Table 2). External validation was most often performed by means of temporal validation (*n* = 15; 25%), in which the validation population is sampled from another time period than the development cohort. Some measure of model performance was reported in nearly all studies (*n* = 57; 95%). Discrimination was reported in 38 studies (63%), typically using the C-statistic (*n* = 36; 95%). Calibration was reported in 19 studies, most often by means of a goodness-of-fit test (Hosmer-Lemeshow) (*n* = 7; 37%). Fourteen studies (23%) reported performance measures on both discrimination and calibration, 24 studies (40%) reported on discrimination alone, five studies (8%) on calibration alone and two on clinical usefulness (3%).

**Table 2:** Summary of Model Characteristics

	Development only with or without internal validation (*n* = 40 studies)	Development and external validation (*n* = 15 studies)	External validation (*n* = 5 studies)
Modelling method^*^			
Regression analysis, no. of studies (%)	31 (78%)	13 (87%)	3 (60%)
Artificial intelligence, no. of studies (%)	9 (23%)	5 (33%)	0 (0%)
Other^†^, no. of studies (%)	4 (10%)	0 (0%)	2 (40%)
*Internal validation*			
Split sample, no. of studies (%)	17 (43%)	1 (7%)	–
Bootstrapping or cross-validation, no. of studies (%)	9 (23%)	1 (7 %)	–
None, no. of studies (%)	14 (35%)	13 (87%)	–
*Performance measures*			
Explained variance			
*R*^2^, no. of studies (%)	21 (53%)	5 (33%)	2 (40%)
Discrimination^‡^	24 (60%)	10 (67%)	4 (80%)
C-statistic/AUC, no. of studies (%)	22 (55%)	10 (67%)	4 (80%)
Discrimination slope, no. of studies (%)	2 (5%)	0 (0%)	0 (0%)
Other^§^, no. of studies (%)	2 (5%)	0 (0%)	1 (20%)
Calibration^‖^	9 (23%)	6 (40%)	4 (80%)
Goodness of fit, no. of studies (%)	4 (10%)	3 (20%)	1 (20%)
Calibration plot, no. of studies (%)	0 (0%)	3 (20%)	4 (80%)
Other^¶^, no. of studies (%)	5 (13%)	4 (27%)	1 (20%)
Classification			
Sensitivity/specificity, no. of studies (%)	14 (35%)	7 (47%)	1 (20%)
Clinical usefulness	1 (3%)	1 (7%)	0 (0%)
Net reclassification index, no. of studies (%)	1 (3%)	0 (0%)	0 (0%)
Decision curve, no. of studies (%)	0 (0%)	1 (7%)	0 (0%)

*AUC* area under the receiver operating curve

*As some studies use multiple modelling strategies when presenting multiple models, totals may add up to > 100%

†For example, risk stratification with predefined risk tiers

‡As some studies use multiple measures of discrimination, totals may add up to > 100%

^§^For example, D-statistic, Brier score, Integrated Discrimination Improvement (IDI)

^‖^As some studies use multiple measures of calibration, totals may add up to > 100%

^¶^For example, calibration slope, root mean square of approximation (RMSEA), cost capture

### Comparison with Proprietary Models

Eight (13%) directly compared their developed models with a proprietary model. One of these studies developed a model with a validated C-statistic of 0.84 while the best-performing model in that study, which included proprietary predictors, yielded a C-statistic of 0.86.^31^ Another study which compared several prediction models, both proprietary and non-proprietary, showed C-statistics ranging from 0.71 to 0.76 for models estimating the risk for patients to become part of the top decile of the cost distribution in the subsequent year.^32^

### Risk of Bias Assessment

**Figure 1:**
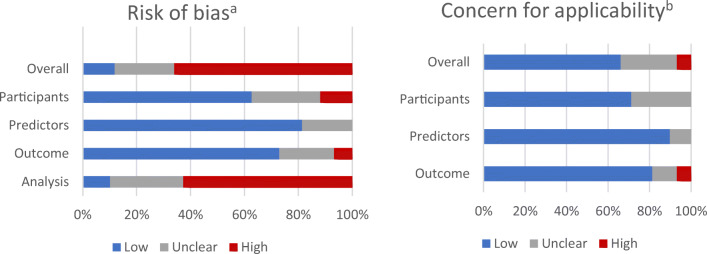
Prediction model Risk Of Bias Assessment Tool (PROBAST) results on risk of bias and concern for applicability in identified models for predicting high-need high-cost healthcare use. (**a**)Risk of bias—assessment whether shortcomings in study design, conduct, or analysis could lead to systematically distorted estimates of a model’s predictive performance (**b**) Concern for applicability—assessment whether the population, predictors, or outcomes of the primary study differ from those specified in the review question.

Risk of bias was rated as ‘*high*’ for 40 studies (67%), ‘*unclear*’ for 13 (22%) and ‘*low*’ for the remaining seven (12%) studies (Fig. 1). Within PROBAST subdomains, most studies (*n* = 37; 62%) scored ‘*high*’ in the ‘*Analysis*’ subdomain. However, risk of bias was ‘*low*’ for the subdomain ‘*Participants*’ in 38 studies (63%), for the subdomain ‘*Predictors*’ in 49 studies (82%) and for the subdomain ‘*Outcome*’ in 45 studies (75%). A full description of PROBAST scores for each study is available in eTable 2 in the Supplement. The high risk of bias in the ‘*Analysis*’ subdomain was mostly due to issues with the handling of missing data (*n* = 50; 83%), limited description of the handling of continuous and categorical predictors (*n* = 48; 80%) and lack of evaluation or reporting of model performance measures (*n* = 44; 73%). Assessment of the applicability of a study to the review question showed overall ‘*low*’ concerns for applicability in 40 studies (67%).

### Applicability for Clinical Use

**Figure 2:**
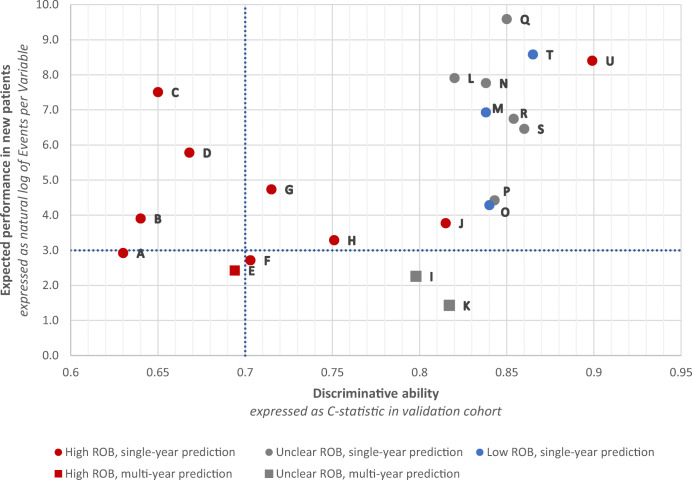
Scatter plot of model performance, indicated by models’ discriminative ability to distinguish those with from those without the outcome (expressed as C-statistic;*X*-axis), vs. models’ expected performance in new patients, indicated by risk of overfitting to the development data (expressed as natural log of EPV, *Y*-axis) and risk of bias (ROB). ^a-d^ (**a**) *X*-axis–C-statistic. (**b**) *Y*-axis—natural log of number of events per variable. (**c**) Horizontal blue line—natural log of EPV 20 (3.0). An EPV ≥ 20 implies minimal risk of overfitting.^26,27^ (**d**) Vertical blue line—C-statistic 0.7. A C-statistic ≥ 0.7 implies good discrimination.^23–25^ (**e**) Risk of bias as assessed through Prediction model Risk Of Bias Assessment Tool (PROBAST).^28^ U = outcome based on utilization; C = outcome based on cost; EPV = events per variable; ROB = risk of bias; ED = emergency department.

Of the 45 studies that performed validation, 17 (38%) did not report a C-statistic and eight (18%) lacked information on candidate variables, resulting in 20 studies (44%) that were assessed for clinical applicability in Figure 2. Two studies presented models predicting multiple outcomes, both on healthcare expenditure and healthcare utilization, and are therefore included in this analysis twice.^33,34^ Among models predicting an expenditure outcome, three showed good discriminative ability, minimal risk of overfitting and low overall risk of bias (*M*, *O and T* in Fig. 2).^31,35,36^ One model (*N* in Fig. 2) showed good discriminative ability and minimal risk of overfitting but had unclear overall risk of bias.^34^ Among models with a predicted outcome on utilization, one showed good discriminative ability and minimal risk of overfitting but an unclear overall risk of bias (*R* in Fig. 2).^34^ Finally, among models with a prediction timespan beyond 12 months, two demonstrated good discriminative ability but showed a risk of overfitting and an unclear risk of bias (*I* and *K* in Fig. 2).^37,38^ External validation of one of these models (HRUPoRT: High Resource User Population Risk Tool) in a separate study demonstrated strong discriminative ability (C-statistic 0.83) and good calibration (*K* in Fig. 2).^37,39^

## DISCUSSION

We provide an overview of non-proprietary models predicting future HNHC healthcare use. In the identified studies, measures of models’ predictive performance in terms of both discrimination and calibration are not consistently provided and external validation is not regularly performed. Most models estimate the risk for patients to become part of a top percentage of the cost-distribution within the next 12 months; only five studies presenting 12 models (8%) had a prediction timespan of more than a year.

Predictors were derived most often from claims data and in 20 studies (33%) from electronic health records (EHR). One explanation may be the ease of data collection from such administrative sources. Another explanation may be that models combining administrative diagnosis data with predictors based on prior cost have been shown to outperform models using only diagnosis or only cost data for predicting individual future high cost.^40^ However, use of administrative data has some disadvantages. Specifically, claims data are often incomplete and precision and utility may be hampered by the potential for patient disenrollment.^14^

Classification according to Andersen’s model showed that none of the studies included predictors from the ‘*Predisposing*’ subcategory beliefs. Andersen and others argued that the absence of beliefs in clinical and health services research may be explained by poor conceptualization and measurement rather than by irrelevance of the concept.^41,42^ Predictors from Andersen’s second category (‘*Enabling*’) were included in only 23 studies (38%), even though prior research emphasized the role of social determinants of health in patients becoming HNHC.^43^ Inclusion of such characteristics is therefore likely to improve selection of patients amenable to intervention. However, ‘*Enabling*’ characteristics such as income or the urban-rural character of a patients’ residence are usually not amenable to clinical intervention whereas the level of illness is. A likely explanation for the underrepresentation of ‘*Enabling*’ predictors in the identified models is the relative unavailability of social determinants in administrative data. Although patient and household interviews are potentially more informative on social determinants, they are costly and time-consuming instruments of data collection with potential for recall bias. Furthermore, not all data sources are available for all study populations. The setting in which case finding is projected is therefore an important aspect in choosing a model because it determines which predictors are accessible and, therefore, which model can be used.

The heterogeneity in predicted outcomes in this review reflects the heterogeneity in HNHC case definition. Some models emphasize high need (e.g. top decile of hospital admissions), while others focus on high cost (e.g. top decile of cost distribution). Yet, modelling high need alone will not fully include high-cost patients and vice versa.^44^ This can be problematic when a model is used as a sole case-finding strategy for HNHC care management. For example, if a patient has a high predicted risk for belonging to next year’s top decile of the cost distribution, this does not inform us on its underlying causes and whether care management is an appropriate intervention.^44^ A more hybrid approach to case finding may be preferred, combining quantitative prediction modelling and an individual, more qualitative assessment, thereby facilitating the inclusion of ‘*Enabling*’ characteristics and beliefs into the selection for care management.^45^

The prediction timespan of a model is another important aspect in applicability in case finding. Although we identified models that predict who will incur high healthcare costs during the subsequent year with a rather high degree of accuracy, patients who remain HNHC over multiple years are particularly interesting candidates to include in care management programmes.^38,46,47^ Previous studies have emphasized that only 28-51% of HNHC patients remained HNHC after a year, whereas others had died or returned to a non-HNHC status.^38,47^ Predicting HNHC persistence rather than HNHC in the next 12 months is therefore more clinically relevant with regard to case finding for HNHC care management. On the other hand, some studies specifically focussed on patients with a temporary HNHC status such as Transient High Utilisers (THUs) in the study by Ng et al. or cost bloomers in the study by Tamang et al. Predicting temporary HNHC status may be particularly difficult but may also provide understanding in the difference between these two populations.^38,46^

Model performance varied and was not always described in detail. Most studies (*n* = 45; 75%) conducted validation, yet this was external in only 20 studies (33%) limiting implications for clinical applicability. All studies described some measure of model performance, but often, this was limited to discrimination alone (*n* = 24; 40%). Prior research has underlined the importance of rigorous validation and performance assessment when developing a prediction model.^22^ However, even in those studies where both discrimination and calibration were described, assessment of clinical usefulness was largely absent.

Comparing models to proprietary models based on summary statistics alone is precarious (e.g. *R*^2^ or the C-statistic). A recent comparative analysis of claims-based tools by the Society of Actuaries, which included 12 models from seven developers, showed that model performance expressed as *R*^2^ varied from 20.5 to 32.1%.^16^ No C-statistics were provided. A comparison of proprietary and non-proprietary models in the same (external validation) sample is much more informative. Studies that directly compared their model(s) with a proprietary model showed relatively comparable discriminative abilities.^31,32^ Clear advantages of proprietary prediction models are that they are thoroughly validated, can be tailored to a specific setting and are usually well-integrated in specific software.^14^ Disadvantages are cost of implementation and the relative lack of transparency with regard to the predictive results.^14^

A high or unclear risk of bias (*n* = 53; 88%) was predominantly due to issues in the ‘*Analysis*’ subdomain of the PROBAST tool. An important reason for risk of bias was the inappropriate handling of continuous or categorical predictors (*n* = 48; 80%), for example, when a continuous predictor was categorized. Although categorization has practical advantages, it leads to an unnecessary loss of information and increases the potential for bias as the categories can be chosen favourably.^48^ Another reason for risk of bias was the use of missing data as an exclusion criterion (*n* = 21; 35%) and limited description of how missing data were handled in general (*n* = 50; 83%). This may lead to selection bias and a potential negative impact on the validity of the model during external validation.^49^

The question which models are most useful for case-finding strategies is best answered in terms of clinical usefulness (i.e. decision-curve analysis). However, this was rarely done in the included studies and is therefore a gap in current evidence.^22,50^ Ideally, a model identifies those patients most amenable to intervention and for whom care management has the potential to improve quality of care while reducing or replacing high-cost care.^13,51^ Various trials demonstrated positive effects of care-management programmes on quality of care, quality of life and healthcare use (e.g. hospitalizations).^7,10,11^ However, evidence is mixed and one of the main reported challenges is the selection of the appropriate population.^7,10,11,45^ Prediction modelling can help identify suitable patients at an early stage. Programmes with a focus on transitional care (i.e. the coordination and continuity of healthcare as patients transfer between different locations or different levels of care) may be served with a model based on hospitalized patients (e.g. *I* in Fig. 2).^52^ On the other hand, an interdisciplinary (primary) care team may be served better by a model based on the general population (e.g. *O or T* in Fig. 2).^35,38,53^ Yet, all these models (*I*, *O and T* in Fig. 2) include predictors on prior cost and therefore require data on prior healthcare cost, which are not always available. Validated models employing techniques other than regression modelling were not included in Figure 2 because an EPV could not be calculated. Yet, this does not imply they are less applicable in practice. For example, one study developed models by means of text mining with good discriminative ability and low overall risk of bias, demonstrating the potential of new modelling techniques.^54^ Thus, the choice of a predictive model, as part of a case-finding strategy for HNHC care management, primarily depends on the setting (e.g. hospital, general population), data availability (e.g. data on prior cost) and specific goals of that programme (e.g. reduction in total cost or number of primary care visits). After considering these aspects, choosing models can be refined based on risk of bias of the study or risk of overfitting.

This study has several limitations. First, a meta-analysis could not be performed because study populations and outcomes vary considerably. Second, studies with populations defined by a single specific disease or condition were excluded (e.g. COPD or Alzheimer) because of focus on models with the potential to be implemented in care-management programmes aimed at a broad target population. However, disease-specific models may provide useful insight into underlying mechanisms of becoming HNHC and may also improve predictive abilities within subpopulations.

This study has several strengths. To our knowledge, this is the first systematic review of non-proprietary prognostic models predicting HNHC healthcare use. Furthermore, we provide practical guidance on how to choose between available models as part of a case-finding strategy for HNHC care-management programmes.

One implication of this review is that, when designing a case-finding strategy for HNHC care management, the most important aspects to consider in choosing a prediction model are setting, data availability and specific goals (i.e. desired outcome) of that programme. Furthermore, future research may benefit from a shift of developing new models to validating and extending existing models.^55^ Another relevant observation is a relative paucity of models predicting HNHC persistence over multiple years, with important implications in identification of suitable candidates for related care-management programmes, often with long-term horizons. On the other hand, one may argue that specific strategies can be used optionally to prevent either transient or persistent HNHC. Furthermore, incorporating models into clinical practice warrants further research, such as decision-curve analyses to improve assessment of clinical usefulness. Lastly, a hybrid approach of quantitative prediction modelling and individual qualitative assessment may improve case finding of appropriate patients for care-management, by specifically including ‘*Beliefs*’ and ‘*Enabling*’ characteristics, such as socioeconomic and psychosocial circumstances.^45^

## CONCLUSIONS

In summary, a variety of models predicting future HNHC healthcare use is available, most often estimating the risk for patients to become part of some top percentage of the cost distribution in the subsequent year. Future research on case-finding strategies for HNHC care-management programmes should focus on validating and extending existing models, develop models that predict HNHC persistence and assess clinical usefulness in order to improve quality of care for this complex patient population.

## Supplementary Information

ESM 1 (DOCX 244 kb)

ESM 2 (DOCX 19 kb)

## References

[1] Wammes JJG, van der Wees PJ, Tanke MAC, Westert GP, Jeurissen PPT (2018). Systematic review of high-cost patients' characteristics and healthcare utilisation. BMJ Open..

[2] Tanke MA, Feyman Y, Bernal-Delgado E (2019). A challenge to all. A primer on inter-country differences of high-need, high-cost patients. PloS one.

[3] Wammes JJG, Tanke M, Jonkers W, Westert GP, Van der Wees P, Jeurissen PP (2017). Characteristics and healthcare utilisation patterns of high-cost beneficiaries in the Netherlands: a cross-sectional claims database study. BMJ Open..

[4] Blumenthal D, Chernof B, Fulmer T, Lumpkin J, Selberg J (2016). Caring for high-need, high-costpatients—an urgent priority. N Engl J Med..

[5] Lee JY, Muratov S, Tarride J, Holbrook AM (2018). Managing High-Cost Healthcare Users: The International Search for Effective Evidence-Supported Strategies. J Am Geriatr Soc..

[6] Baker JM, Grant RW, Gopalan A (2018). A systematic review of care management interventions targeting multimorbidity and high care utilization. BMC health services research..

[7] Bleich SN, Sherrod C, Chiang A (2015). Systematic Review of Programs Treating High-Need and High-Cost People With Multiple Chronic Diseases or Disabilities in the United States, 2008-2014. Prev Chronic Dis..

[8] Ouwens M, Wollersheim H, Hermens R, Hulscher M, Grol R (2005). Integrated care programmes for chronically ill patients: a review of systematic reviews. International journal for quality in health care..

[9] Brown RS, Peikes D, Peterson G, Schore J, Razafindrakoto CM (2012). Six features of Medicare coordinated care demonstration programs that cut hospital admissions of high-risk patients. Health Aff..

[10] **Hong CS, Siegel AL, Ferris TG**. Caring for high-need, high-cost patients: what makes for a successful care management program?. 2014. 25115035

[11] McCarthy D, Ryan J, Klein S (2015). *Models of care for high-need, high-cost patients: an evidence synthesis*.

[12] **Lewis G, Curry N, Bardsley M**. Choosing a predictive risk model: a guide for commissioners in England*. London: Nuffield Trust*. 2011;20.

[13] **Davis AC, Osuji TA, Chen J, Lyons LJL, Gould MK**. Identifying populations with complex needs: variation in approaches used to select complex patient populations*. Population Health Management*. 2020. 10.1089/pop.2020.0153 32941105

[14] Hwang AS (2015). *Finding a match: how successful complex care programs identify patients*.

[15] **Cumming RB, Knutson D, Cameron BA, Derrick B.** A comparative analysis of claims-based methods of health risk assessment for commercial populations*. Final report to the Society of Actuaries*. 2002.

[16] **Winkelman R, Mehmud S**. A comparative analysis of claims-based tools for health risk assessment*. Society of Actuaries*. 2007:1-70.

[17] Moher D, Liberati A, Tetzlaff J, Altman DG (2010). Preferred reporting items for systematic reviews and meta-analyses: the PRISMA statement. Int J Surg..

[18] Rethlefsen ML, Kirtley S, Waffenschmidt S (2021). PRISMA-S: an extension to the PRISMA statement for reporting literature searches in systematic reviews. Systematic reviews..

[19] Reynders RM, Ladu L, Di Girolamo N (2019). Contacting of authors modified crucial outcomes of systematic reviews but was poorly reported, not systematic, and produced conflicting results. J Clin Epidemiol..

[20] **Andersen R, Newman JF**. Societal and individual determinants of medical care utilization in the United States*. Milbank Q*. 2005;83(4):Online-only. 4198894

[21] Steyerberg EW, Vickers AJ, Cook NR (2010). Assessing the performance of prediction models: a framework for some traditional and novel measures. Epidemiology..

[22] Steyerberg EW, Vergouwe Y (2014). Towards better clinical prediction models: seven steps for development and an ABCD for validation. Eur Heart J..

[23] Steyerberg EW, Vickers AJ, Cook NR (2010). Assessing the performance of prediction models: a framework for some traditional and novel measures. Epidemiology..

[24] Pencina MJ, D’Agostino RB (2015). Evaluating discrimination of risk prediction models: the C statistic. JAMA..

[25] Hosmer DW, Lemeshow S (2000). Applied Logistic Regression.

[26] Peduzzi P, Concato J, Kemper E, Holford TR, Feinstein AR (1996). A simulation study of the number of events per variable in logistic regression analysis. J Clin Epidemiol..

[27] van Smeden M, de Groot JA, Moons KG (2016). No rationale for 1 variable per 10 events criterion for binary logistic regression analysis. BMC Medical Research Methodology..

[28] Wolff RF, Moons KG, Riley RD (2019). PROBAST: a tool to assess the risk of bias and applicability of prediction model studies. Ann Intern Med..

[29] **Rosella LC, Kornas K, Sarkar J, Fransoo R**. External Validation of a Population-Based Prediction Model for High Healthcare Resource Use in Adults. *Healthcare*. 2020;8(4):537. 10.3390/healthcare8040537 PMC7761789 33291559

[30] Rosella LC, Kornas K, Yao Z (2018). Predicting high health care resource utilization in a single-payer public health care system: development and validation of the high resource user population risk tool. Med Care..

[31] Rakovski CC, Rosen AK, Wang F, Berlowitz DR (2002). Predicting elderly at risk of increased future healthcare use: How much does diagnostic information add to prior utilization?. Health Serv Outcomes Res..

[32] Haas LR, Takahashi PY, Shah ND (2013). Risk-stratification methods for identifying patients for care coordination. Am J Manag Care..

[33] Wherry LR, Burns ME, Leininger LJ (2014). Using Self-Reported Health Measures to Predict High-Need Cases among Medicaid-Eligible Adults. Health Serv Res..

[34] **Orueta JF, García-Alvarez A, Aurrekoetxea JJ, García-Goñi M**. FINGER (Forming and Identifying New Groups of Expected Risks): developing and validating a new predictive model to identify patients with high healthcare cost and at risk of admission*. BMJ open*. 2018;8(5). 10.1136/bmjopen-2017-019830 PMC5988109 29858409

[35] Osawa I, Goto T, Yamamoto Y, Tsugawa Y (2020). Machine-learning-based prediction models for high-need high-cost patients using nationwide clinical and claims data. NPJ digital medicine..

[36] Chechulin Y, Nazerian A, Rais S, Malikov K (2014). Predicting patients with high risk of becoming high-cost healthcare users in Ontario (Canada). Healthc Policy..

[37] Rosella LC, Kornas K, Yao Z (2018). Predicting high health care resource utilization in a single-payer public health care system: development and validation of the high resource user population risk tool. Med Care..

[38] Ng SHX, Rahman N, Ang IYH (2020). Characterising and predicting persistent high-cost utilisers in healthcare: a retrospective cohort study in Singapore. BMJ open..

[39] **Rosella LC, Kornas K, Sarkar J, Fransoo R**. External Validation of a Population-Based Prediction Model for High Healthcare Resource Use in Adults. 2020;8(4):537. 10.3390/healthcare8040537 PMC7761789 33291559

[40] Ash AS, Zhao Y, Ellis RP, Schlein KM (2001). Finding future high-cost cases: comparing prior cost versus diagnosis-based methods. Health Serv Res..

[41] **Andersen RM**. Revisiting the behavioral model and access to medical care: does it matter?*. J Health Soc Behav*. 1995:1-10. 7738325

[42] **Becker MH, Maiman LA**. *'Models of Health-Related Behavior' in Handbook of Health, Health Care,and the Profession .* New York: The Free Press.

[43] Fitzpatrick T, Rosella LC, Calzavara A (2015). Looking beyond income and education: socioeconomic status gradients among future high-cost users of health care. Am J Prev Med..

[44] Kieu Nguyen O, Tang N, Hillman JM, Gonzales R (2013). What's cost got to do with it? Association between hospital costs and frequency of admissions among “high users” of hospital care. Journal of hospital medicine..

[45] Haime V, Hong C, Mandel L (2015). Clinician considerations when selecting high-risk patients for care management. Am J Manag Care..

[46] Tamang S, Milstein A, Sørensen HT (2017). Predicting patient ‘cost blooms’ in Denmark: a longitudinal population-based study. BMJ open..

[47] Johnson TL, Rinehart DJ, Durfee J (2015). For many patients who use large amounts of health care services, the need is intense yet temporary. Health Aff..

[48] Bennette C, Vickers A (2012). Against quantiles: categorization of continuous variables in epidemiologic research, and its discontents. BMC medical research methodology..

[49] **Hernán MA, Hernández-Díaz S, Robins JM**. A structural approach to selection bias*. Epidemiology*. 2004:615-625. 10.1097/01.ede.0000135174.63482.43 15308962

[50] Fitzgerald M, Saville BR, Lewis RJ (2015). Decision curve analysis. JAMA..

[51] Murphy SM, Castro HK, Sylvia M (2011). Predictive modeling in practice: improving the participant identification process for care management programs using condition-specific cut points. Population health management..

[52] Coleman EA, Berenson RA (2004). Lost in transition: challenges and opportunities for improving the quality of transitional care. Ann Intern Med..

[53] Chechulin Y, Nazerian A, Rais S, Malikov K (2014). Predicting patients with high risk of becoming high-cost healthcare users in Ontario (Canada). Healthc Policy..

[54] Frost DW, Vembu S, Wang J, Tu K, Morris Q, Abrams HB (2017). Using the Electronic Medical Record to Identify Patients at High Risk for Frequent Emergency Department Visits and High System Costs. Am J Med..

[55] Bayerstadler A, Benstetter F, Heumann C, Winter F (2014). A predictive modeling approach to increasing the economic effectiveness of disease management programs. Health Care Manag Sci..

