# Supplementary material for: Prediction Models for Future High-Need High-Cost Healthcare Use: a Systematic Review
Source: J Gen Intern Med. 2022 Jan 11;37(7):1763–70. doi: 10.1007/s11606-021-07333-z (PMC9130365; doi:10.1007/s11606-021-07333-z)
Supplement: Supplementary file 1 — (DOCX 244 kb) [file 11606_2021_7333_MOESM1_ESM.docx]

**APPENDIX**

eMethods Description of search strategies, study selection, data extraction and synthesis

eFigure 1 Preferred Reporting Items for Systematic Reviews and Meta-Analyses (PRISMA) flow diagram of study selection

eTable 1 Characteristics of included studies

eTable 2 Prediction model Risk Of Bias Assessment Tool (PROBAST) results

eTable 3 Summary of findings on contacting authors of eligible studies

eReferences

**eMethods**

**Search strategies**

The search strategies were determined in collaboration with a systematic review specialist. The databases of Ovid MEDLINE® All, EMBASE (via Embase.com), CINAHL (EBSCOhost), Web of Science Core Collection and Google Scholar from inception through January 31^st^ 2021. No filters for language, publishing date or study design were applied. The search was rerun on the 31^st^ of January 2021 before finalizing the manuscript. An email alert on new studies has been in place since. References of identified articles were searched by hand for relevant articles.

Embase.com (1971-) 4183 (4254)

((('health care utilization'/exp OR 'hospital utilization'/de) AND 'health care cost'/exp) OR (((frequent* OR high OR super* OR high-resource*) NEXT/1 (users OR user OR consult* OR utili* OR visit* OR flyer OR resource* OR attend* OR spend*)) OR Medical-Overuse OR superutili* OR overutili* OR ((high-need or high-cost) NEXT/1 (patient* OR people* OR user* OR healthcare-user* OR health-care-user* OR utili* OR case*)) OR ((Upper OR top) NEXT/1 decile NEAR/3 expend*) OR (((health-care OR high-care OR health-service OR healthcare OR resource* OR medical) NEAR/3 (utili* OR use)) AND ((high*) NEAR/3 (cost OR costs))) OR ((top-20 OR top-10 OR top-5 OR percentile*) NEAR/15 (cost* OR expend* OR users OR utili* OR spend*))):ab,ti,kw OR (high* AND (utili* OR future* OR healthcare OR health-care) AND (cost OR costs OR expend*)):ti) AND ((('prediction'/exp OR 'regression analysis'/exp OR forecasting/de OR 'predictive value'/de OR 'validation study'/de OR 'risk assessment'/de OR 'high risk patient'/de OR 'patient coding'/de) AND ('model'/de OR 'theoretical model'/de OR 'statistical model'/de OR 'machine learning'/de OR 'artificial intelligence'/de)) OR 'prediction'/exp/mj OR forecasting/mj OR (((predict* OR regress* OR future* OR risk* OR forecast*) NEAR/10 (model* OR high*-cost OR machine*-learn* OR artificial*-intelligen*))):ab,ti,kw OR (predict* OR forecast* OR identif* OR finding* OR ((Derivat* OR Validat* OR develop* OR validat*) AND Model*)):ti) NOT ((juvenile/exp OR pediatrics/exp OR (child* OR infan* OR pediatric* OR paediatric*):ab,ti,kw) NOT (adult/exp OR (adult*):ab,ti,kw))

Medline ALL Ovid (1946-) 173 (1716)

(((Patient Acceptance of Health Care /) AND Health Care Costs /) OR (((frequent* OR high OR super* OR high-resource*) ADJ (users OR user OR consult* OR utili* OR visit* OR flyer OR resource* OR attend* OR spend*)) OR Medical-Overuse OR superutili* OR overutili* OR ((high-need or high-cost) ADJ (patient* OR people* OR user* OR healthcare-user* OR health-care-user* OR utili* OR case*)) OR ((Upper OR top) ADJ decile ADJ3 expend*) OR (((health-care OR high-care OR health-service OR healthcare OR resource* OR medical) ADJ3 (utili* OR "use")) AND ((high*) ADJ3 (cost OR costs))) OR ((top-20 OR top-10 OR top-5 OR percentile*) ADJ15 (cost* OR expend* OR users OR utili* OR spend*))).ab,ti,kw. OR (high* AND (utili* OR future* OR healthcare OR health-care) AND (cost OR costs OR expend*)).ti.) AND (((Regression Analysis/ OR Forecasting/ OR Validation Studies/ OR Risk Assessment/) AND (model/ OR Models, Theoretical/ OR Models, Statistical/ OR axp Machine Learning/ OR Artificial Intelligence/)) OR *Forecasting/ OR (((predict* OR regress* OR future* OR risk* OR forecast*) ADJ10 (model* OR high*-cost OR machine*-learn* OR artificial*-intelligen*))).ab,ti,kw. OR (predict* OR forecast* OR identif* OR finding* OR ((Derivat* OR Validat* OR develop* OR validat*) AND Model*)).ti.) NOT ((exp children/ OR pediatrics/ OR (child* OR infan* OR pediatric* OR paediatric*).ab,ti,kw.) NOT (exp adults/ OR (adult*).ab,ti,kw.))

Web of Science Core Collection (1975-) 1427 (2733)

((TS=(((frequent* OR high OR super* OR high-resource*) NEAR/1 (users OR user OR consult* OR utili* OR visit* OR flyer OR resource* OR attend* OR spend*)) OR Medical-Overuse OR superutili* OR overutili* OR ((high-need or high-cost) NEAR/1 (patient* OR people* OR user* OR healthcare-user* OR health-care-user* OR utili* OR case*)) OR ((Upper OR top) NEAR/1 decile NEAR/2 expend*) OR (((health-care OR high-care OR health-service OR healthcare OR resource* OR medical) NEAR/2 (utili* OR use)) AND ((high*) NEAR/2 (cost OR costs))) OR ((percentile*) NEAR/15 (cost* OR expend* OR users OR utili* OR spend*))) OR TI=(high* AND (utili* OR future* OR healthcare OR health-care) AND (cost OR costs OR expend*))) AND (TS=(((predict* OR regress* OR future* OR risk* OR forecast*) NEAR/10 (model* OR high-cost OR machine-learn* OR artificial-intelligen*))) OR TI=(predict* OR forecast* OR identif* OR finding* OR ((Derivat* OR Validat* OR develop* OR validat*) AND Model*))) NOT TS=(((child* OR infan* OR pediatric* OR paediatric*)) NOT ((adult*)))) AND TS=(hospital* OR health-care OR healthcare OR therap* OR disease* OR patient* OR medical* OR clinical*)

**Study selection**

The screening process was performed through Covidence systematic review software.^68^ During the process the reviewers were blinded to each other’s decisions. In case of discrepancy, a third author (HL) was consulted. Studies regarding proprietary models were considered beyond the scope of this review as these tools have been described and reviewed in detail elsewhere.^15, 16^ Furthermore, studies regarding prediction of Emergency Room (ER) readmission within a timespan of 30 days were also considered beyond the scope of this review as these have also been described and reviewed in detail elsewhere and are not logical tools as part of a case-finding strategy for long-term HNHC care management.^69, 70^

**Predetermined in- and exclusion criteria**

1. Is the full text of the article in English?

Yes Proceed to #2

No Code EXCLUDE1 and STOP

2. Is the article a literature review (systematic or otherwise), meta-analysis, letter to the editor or methodologic study only?

Yes Code EXCLUDE2 and STOP

NO Proceed to #3

3. Does the study population include only adults?

Yes Proceed to #4

No Code EXCLUDE3 and STOP`

4. Is the base population of the study defined by a specific disease or morbidity?

Yes Code EXCLUDE4 and STOP

No Proceed to #5

5. Is the article a primary study that develops, validates or extends a prognostic prediction model with a time horizon beyond the index event (i.e. a prognostic prediction model)?

Yes Proceed to #6

No Code EXCLUDE5 and STOP

6. Is the outcome of the prognostic prediction model a measure of High-Need High-Cost healthcare use with a cost component and is this explicitly described? (e.g. top-x% of future healthcare cost, measure of utilization etc)

Yes Proceed to #7

No Code EXCLUDE6 and STOP

7. Does the study solely concern a proprietary prediction model?

Yes Code EXCLUDE7 and STOP

No Include

Base population: General adult population

Intervention: Prognostic prediction models derived and/or validated in a cohort of adults

Comparator: Not applicable

Outcome: High-Need High-Cost healthcare use defined with at least a relative or absolute measure of cost and excluding Emergency Room readmission within 30 days as sole outcome

Timing: Beyond 30 days after the index event

**Data extraction**

Data-extraction was performed according to the CHecklist for critical Appraisal and data extraction for systematic Reviews of prediction Modeling Studies (CHARMS) and Transparent Reporting of a multivariable prediction model for Individual Prognosis Or Diagnosis (TRIPOD).^71, 72^ From each study we extracted the following data: population characteristics, sample size in development and (if applicable) validation population, population setting, outcome definition, candidate and final predictors and model performance measures.

### Andersen’s Behavioural Model of Healthcare Utilization

All final predictors were categorized according to Andersen’s Behavioural Model of Healthcare Utilization. As the aim of our review is to assess their applicability as part of a case-finding strategy for clinical HNHC care-management programs, we focused on individual determinants of health services utilization rather than delivery system characteristics or social norms on illness. Table 1 details the different (sub)categories of individual predictors.

| **PREDISPOSING** | | **ENABLING** | | | **ILLNESS LEVEL** | |
| --- | --- | --- | --- | --- | --- | --- |
| Characteristics which exist prior to the onset of specific episodes of illness that can predict propensity toward use of health services | | Conditions which permit a family to act on a value or satisfy a need regarding health service use | | | The perception of illness or the probability of its occurrence by the individual (or his family) and by clinical evaluation | |
|  | |  | | |  | |
| **1) Demographic** | | **1) Family** | | | **1) Perceived illness level** | |
|  | *Age* |  | *Income* | |  | *Disability* |
|  | *Sex* |  | *Health insurance* | |  | *Symptoms* |
|  | *Marital status* |  | *Type of regular source* | |  | *Diagnoses* |
|  | *Past illness* |  | *Access to regular source* | |  | *General state* |
|  |  |  |  | |  |  |
| **2) Social Structure** | | **2) Community** | | | **2) Evaluated illness level** | |
|  | *Education* |  | | *Ratios of health personnel and facilities to population* |  | *Symptoms* |
|  | *Race* |  | | *Price of health services* |  | *Diagnoses* |
|  | *Occupation* |  | | *Region of county* |  | |
|  | *Family size* |  | | *Urban-rural character* |  | |
|  | *Ethnicity* |  | | |  | |
|  | *Religion* |  | | |  | |
|  | *Residential mobility* |  | | |  | |
|  |  |  | | |  | |
| **3) Beliefs** | |  | | |  | |
|  | *Values concerning health and illness* |  | | |  | |
|  | *Attitudes towards health services* |  | | |  | |
|  | *Knowledge about disease* |  | | |  | |

eMethods Table 1 – Individual determinants of health service utilization according to Andersen’s behavioural model of healthcare utilization. Adapted from “Societal and individual determinants of medical care utilization in the United States” by R. Andersen and J.F. Newman, 2005, *The Milbank Quarterly*, *83*(4).^20^

**Model performance measures**

Discrimination, defined as the accuracy of predictions to discriminate between those with and those without the outcome, was assessed with the Area Under the Receiver Operating Characteristic-Curve (AUC or C-statistic). The C-statistic measures the concordance between model-based risk estimates and observed events. A C-statistic of 0.5 implies that the prediction model is performing no better than random chance, while 1.0 implies prefect prediction.^21^ Other measures of discrimination (Brier score, discrimination slope) have been proposed as more informative alternatives to the C-statistic.^24^ However, it is one of the most common measures used and in that context does provide insight in potential differences between models.^21, 24^ Evaluation of model performance also included the reported measures of calibration and classification. Calibration describes the agreement between observed and predicted outcomes.^21, 28^ A graphical assessment can be made by a calibration plot which has an associated intercept (amount that predictions are consistently over- or underestimated) and a calibration slope.^21, 28^ Another measure of calibration is the Hosmer-Lemeshow goodness-of-fit test.^21^ Additionally, an indication of overall model performance is often provided in terms of explained variance which describes the proportion of variation that a model accounts for, usually by means of an R2.^21^

Another way of judging model performance is assessing the expected performance of a model in new patients by looking at the risk of overfitting to the development data. The ratio between the number of events relative to the number of candidate variables (or more precisely the number of regression coefficients) is known as the number of events per variable (EPV).^27, 73, 74^ For a long time an EPV of ≥ 10 was considered to minimize the risk of overfitting.^27, 73, 74^ However, recent studies have called this into question and proposed a minimum of twenty.^27, 73, 74^ In PROBAST this issue is addressed in the ‘*Analysis’* subdomain with signalling question 4.1: ‘*Were there a reasonable number of participants with the outcome?*’.^28^ PROBAST adheres to a minimum EPV of 20 and leaves EPVs between 10 and 20 up for judgement based on the outcome frequency, overall model performance, and distribution of the predictors in the model.^27, 28^

In Figure 2 we assessed clinical usefulness of all validated models by plotting model performance (expressed as C-statistic) against the risk of overfitting to the development data (expressed as natural log of the events per variable (EPV)). For readability purposes of the scatter plot we used the natural log of EPV rather than EPV itself due to the large range.

**eFigure 1. Preferred Reporting Items for Systematic Reviews and Meta-Analyses (PRISMA) flow diagram of study selection**

**Identification of studies via other methods**

**Identification of studies via databases and registers**

Records identified from:

Citation searching (n = 87)

Records removed *before screening*:

Duplicate records removed (n = 2,900)

Records identified from:

Embase (n = 4,254)

Medline (n = 1,716)

Web of Science (n = 2,733)

**Identification**

Records excluded

(n = 5,360)

Records screened

(n = 5,803)

Reports not retrieved

(n = 5)

Reports sought for retrieval

(n = 87)

Reports not retrieved

(n = 69)

Reports sought for retrieval

(n = 443)

**Screening**

Reports excluded (n = 67):

Disease specific population (n = 32)

No prognostic prediction model (n = 24)

Review / meta-analysis / letter (n = 7)

Proprietary model only (n = 4)

Reports excluded (n = 329):

Disease specific population (n = 158)

No prognostic prediction model (n = 111)

Proprietary model only (n = 26)

Review / meta-analysis / letter (n = 19)

Outcome no measure of HNHC (n = 7)

Not in English (n = 4)

Not adults only (n = 4)

Reports assessed for eligibility

(n = 82)

Reports assessed for eligibility

(n = 374)

Studies included in review

(n = 60)

**Included**

**eTable 1. Characteristics included studies**

| **Study**  First author  Year of publication  Country of publication  Development  Validation (Internal / External) | **Number of Unique Models** | **Pro- or Retrospective Data** | **Base Population Setting** | **Outcome**  Outcome Category  - Healthcare expenditure  - Healthcare utilization  - Clinical outcomes  Study definition of outcome | **Sample Size**  Development cohort  Validation cohort  (Participants with outcome) | **Type of Final Predictors**  - Demographics  - Social Structure  - Beliefs  - Enabling Family  - Enabling Community  - Perceived Need  - Evaluated Need  - Prior utilization | **Longest** **Prediction Timespan** | **Performance Measures**  - Sensitivity / specificity  - Explained variance  - Discrimination  - Calibration  - Classification  *-* Other |
| --- | --- | --- | --- | --- | --- | --- | --- | --- |
| AlSnih^75^  2006  USA  D | 3 | R | Insured general population | Healthcare utilization  1) Physician utilization (no. doctor visits in 12 months  2) Hospital utilization (hospitalization and no. of hospital days) | D 1987 (1709; 375) | Demographics  Social Structure  Enabling Family  Evaluated Need | 12 | Explained Variance |
| Anderson^76^  2004  USA  D+IV | 2 | P | Medicare | Healthcare expenditure  1) Classification into cost groups: top 10% (very high), top 10-20% (high), top 20-30% (medium), and 40% (low) | D 7829 (783; 783; 783; 5480)  V 3915 (392; 392; 392; 2739) | Demographics  Perceived Need  Evaluated Need  Prior utilization | 12 | Sensitivity / specificity  Classification |
| Bertakis^77^  2010  USA  D | 5 | R | Primary care | Healthcare expenditure  1) Annualized medical center resource use | D 509 (509) | Demographics  Social Structure  Enabling Family  Perceived Need  Evaluated Need | 12 | Explained Variance |
| Billings^78^  2006  UK  D+EV | 1 | R | Hospitalized | Healthcare utilization  1) Hospital admission risk score at threshold 50, 70 and 80 | D NA (17455; 4810; 2011)  V NA (NA) | Demographics  Social Structure  Prior utilization | 12 | Sensitivity / Specificity  Discrimination |
| **Study**  First author  Year of publication  Country of publication  Development  Validation (Internal / External) | **Number of Unique Models** | **Pro- or Retrospective Data** | **Base Population Setting** | **Outcome**  Outcome Category  - Healthcare expenditure  - Healthcare utilization  - Clinical outcomes  Study definition of outcome | **Sample Size**  Development cohort  Validation cohort  (Participants with outcome) | **Type of Final Predictors**  - Demographics  - Social Structure  - Beliefs  - Enabling Family  - Enabling Community  - Perceived Need  - Evaluated Need  - Prior utilization | **Longest** **Prediction Timespan** | **Performance Measures**  - Sensitivity / specificity  - Explained variance  - Discrimination  - Calibration  - Classification  *-* Other |
| Blumenthal^79^  2017  USA  D | 10 | R | Insured general population | Healthcare utilization  1) Top 5% ED visits  2) Top 5% hospitalizations | D 2638 (132; 132) | Demographics  Enabling Family  Enabling Community  Perceived Need  Prior utilization | 12 | Sensitivity / Specificity |
| Bosworth^80^  2000  USA  D | 2 | P | VA healthcare service | Healthcare utilization  1) High care use defined as >12 outpatient clinic visits  2) Low-care use as ≤12 outpatient clinic visits | D 136 (NA) | Demographics  Social Structure  Perceived Need | 12 | Discrimination  Calibration |
| Boult^81^  2004  USA  D+EV | 1 | R | Insured general population | Healthcare expenditure  1) Top 10% of total annualized healthcare expenditures | D 827 (83)  V 1027 (103)  V 903 (90) | Demographics  Perceived Need  Evaluated Need  Prior utilization | 12 | Explained Variance  Other (Cost Ratio) |
| Chang^82^  2002  Taiwan  D+IV | 5 | R | Taiwanese population | Healthcare expenditure  1) Total healthcare expenditure  2) Total outpatient expenditure  3) Total inpatient expenditure | D 180451 (180451)  V 179586 (179586) | Demographics  Evaluated Need  Prior utilization | 12 | Explained Variance |
| **Study**  First author  Year of publication  Country of publication  Development  Validation (Internal / External) | **Number of Unique Models** | **Pro- or Retrospective Data** | **Base Population Setting** | **Outcome**  Outcome Category  - Healthcare expenditure  - Healthcare utilization  - Clinical outcomes  Study definition of outcome | **Sample Size**  Development cohort  Validation cohort  (Participants with outcome) | **Type of Final Predictors**  - Demographics  - Social Structure  - Beliefs  - Enabling Family  - Enabling Community  - Perceived Need  - Evaluated Need  - Prior utilization | **Longest** **Prediction Timespan** | **Performance Measures**  - Sensitivity / specificity  - Explained variance  - Discrimination  - Calibration  - Classification  *-* Other |
| Charlson^83^  2014  USA  D+IV | 6 | R | Health and Hospital Union workers | Healthcare expenditure  1) Top 10% of total annualized healthcare expenditures | D 181764 (18178)  V 90882 (9088) | Demographics  Evaluated Need  Prior utilization | 12 | Sensitivity / Specificity  Explained Variance  Discrimination |
| Charlson^84^  2008  USA  D+IV | 1 | P | Primary care | Healthcare expenditure  1) Total annualized healthcare expenditures | D 2931 (2931)  V 2930 (2930) | Demographics  Evaluated Need | 12 | Explained Variance |
| Chechulin^36^  2014  Canada  D+EV | 1 | R | Ontario Residents | Healthcare expenditure  1) Top 5% of total annualized healthcare expenditures | D 10300856 (515043)  V 10185978 (509299) | Demographics  Social Structure  Enabling Community  Evaluated Need  Prior utilization | 12 | Sensitivity / Specificity  Discrimination  Calibration |
| Cohen^85^  2006  USA  D+EV | 2 | R | Insured general population | Healthcare expenditure  1) Top 10% of total annualized healthcare expenditures | D 9810 (981)  V 10129 (1013) | Demographics  Social Structure  Enabling Family  Enabling Community  Perceived Need  Prior utilization | 12 | Sensitivity / Specificity  Discrimination  Calibration |
| **Study**  First author  Year of publication  Country of publication  Development  Validation (Internal / External) | **Number of Unique Models** | **Pro- or Retrospective Data** | **Base Population Setting** | **Outcome**  Outcome Category  - Healthcare expenditure  - Healthcare utilization  - Clinical outcomes  Study definition of outcome | **Sample Size**  Development cohort  Validation cohort  (Participants with outcome) | **Type of Final Predictors**  - Demographics  - Social Structure  - Beliefs  - Enabling Family  - Enabling Community  - Perceived Need  - Evaluated Need  - Prior utilization | **Longest** **Prediction Timespan** | **Performance Measures**  - Sensitivity / specificity  - Explained variance  - Discrimination  - Calibration  - Classification  *-* Other |
| Cunningham^86^  2017  USA  D | 6 | P | Privately insured & Medicaid | Healthcare expenditure  1) Top 25% of total annualized healthcare expenditures | D 3983 (996) | Demographics  Social Structure  Enabling Family  Enabling Community  Perceived Need  Prior utilization | 12 | Sensitivity / Specificity  Explained Variance  Discrimination |
| DeSalvo^57^  2005  USA  D | 21 | P | VA healthcare service | Healthcare utilization  1) Hospitalization (dichotomous)  2) High use of outpatient services (top 10% of total visits)  Clinical outcome  3) Mortality | D 21762 (21762; 2176) | Demographics  Social Structure  Perceived Need  Evaluated Need  Prior utilization | 12 | Calibration  Discrimination |
| DeSalvo^63^  2009  USA  D+EV | 20 | P | Privately insured, Medicare & Medicaid | Healthcare expenditure  1) Total annualized healthcare expenditures (Top 5, 10 and 25%)  2) Annualized pharmacy expenditures (Top 5, 10 and 25%) | D 7948 (397; 795; 1987)  V 7921 (396; 792; 1980) | Demographics  Perceived Need | 12 | Sensitivity / Specificity  Discrimination |
| **Study**  First author  Year of publication  Country of publication  Development  Validation (Internal / External) | **Number of Unique Models** | **Pro- or Retrospective Data** | **Base Population Setting** | **Outcome**  Outcome Category  - Healthcare expenditure  - Healthcare utilization  - Clinical outcomes  Study definition of outcome | **Sample Size**  Development cohort  Validation cohort  (Participants with outcome) | **Type of Final Predictors**  - Demographics  - Social Structure  - Beliefs  - Enabling Family  - Enabling Community  - Perceived Need  - Evaluated Need  - Prior utilization | **Longest** **Prediction Timespan** | **Performance Measures**  - Sensitivity / specificity  - Explained variance  - Discrimination  - Calibration  - Classification  *-* Other |
| DeSalvo^63^  2009  USA  (continued) |  |  |  | 3) Annualized office-based provider expenditures (Top 5, 10 and 25%)  4) Any inpatient expenditure |  |  |  |  |
| Dove^87^  2003  USA  D+IV+EV | 1 | R | Members of large HMO | Healthcare expenditure  1) Medical expenses defined as expenditures >2000 and <2000 US dollars | D 104500 (104500)  IV 104500 (104500)  EV 176642 (176642) | Demographics  Prior utilization | 12 | Discrimination |
| Fleishman^88^  2006  USA  D | 12 | R | Insured general population | Healthcare expenditure  1) Per member per month expenditure | D 1087 (1087) | Demographics  Social Structure  Enabling Family  Enabling Community  Perceived Need  Evaluated Need  Prior utilization | 1 | Calibration  Explained Variance  Other (Cooks D-statistic) |
| Fleishman^62^  2010  USA  D+EV | 6 | R | Insured general population | Healthcare expenditure  1) Top 10% of total annualized healthcare expenditures | D 52918 (5292)  V 61155 (6116) | Demographics  Perceived Need  Evaluated Need | 12 | Sensitivity / Specificity  Explained Variance  Discrimination  Calibration  Other (BIC) |
| **Study**  First author  Year of publication  Country of publication  Development  Validation (Internal / External) | **Number of Unique Models** | **Pro- or Retrospective Data** | **Base Population Setting** | **Outcome**  Outcome Category  - Healthcare expenditure  - Healthcare utilization  - Clinical outcomes  Study definition of outcome | **Sample Size**  Development cohort  Validation cohort  (Participants with outcome) | **Type of Final Predictors**  - Demographics  - Social Structure  - Beliefs  - Enabling Family  - Enabling Community  - Perceived Need  - Evaluated Need  - Prior utilization | **Longest** **Prediction Timespan** | **Performance Measures**  - Sensitivity / specificity  - Explained variance  - Discrimination  - Calibration  - Classification  *-* Other |
| Freedman^56^  1996  USA  D+IV | 1 | R | Insured general population | Healthcare utilization  1) Hospital admission defined as any visit to acute care | D 1873 (1873)  V 1872 (1872) | Demographics  Evaluated Need  Perceived Need | 4.5 | Discrimination  Calibration |
| Frost^54^  2017  Canada  D+IV | 2 | P | Primary care | Healthcare expenditure  1) Top 5% of total annualized healthcare expenditures  Healthcare utilization  2) ≥ 3 or ER visits in subsequent year | D 21680 (1280; 1237)  V 21431 (1072; 855) | Demographics  Social Structure  Enabling Family  Evaluated Need | 12 | Sensitivity / Specificity  Discrimination |
| Haas^32^  2013  USA  V | 3 | R | Primary care | Healthcare utilization  1) Any inpatient hospitalization  2) No. of ED visits not resulting in hospitalizations  3) Any readmission ≤ 30 days  Healthcare expenditure  4) Top 10% of total annualized healthcare expenditures | V 83187 (6858; 10733; 931; 8319) | Demographics  Perceived Need  Evaluated Need  Prior utilization | 12 | Discrimination  Calibration |
| **Study**  First author  Year of publication  Country of publication  Development  Validation (Internal / External) | **Number of Unique Models** | **Pro- or Retrospective Data** | **Base Population Setting** | **Outcome**  Outcome Category  - Healthcare expenditure  - Healthcare utilization  - Clinical outcomes  Study definition of outcome | **Sample Size**  Development cohort  Validation cohort  (Participants with outcome) | **Type of Final Predictors**  - Demographics  - Social Structure  - Beliefs  - Enabling Family  - Enabling Community  - Perceived Need  - Evaluated Need  - Prior utilization | **Longest** **Prediction Timespan** | **Performance Measures**  - Sensitivity / specificity  - Explained variance  - Discrimination  - Calibration  - Classification  *-* Other |
| Harrison^89^  2012  USA  D | 1 | P | Insured general population | Total Healthcare Utilization  1) Inpatient hospital admission  2) ER visits  Healthcare expenditure  3) Total Healthcare Expenditure | D 2245 (2245) | Demographics  Perceived Need  Evaluated Need  Prior utilization | 12 | - |
| Heins^90^  2020  The Netherlands  D | 3 | R | Primary care | Healthcare Utilization  1) ≥12 contacts;  2) ≥1 ER visit  3) ≥ unplanned hospitalisations | D 245065 (245065) | Demographics  Prior utilization | 12 | Discrimination  Sensitivity/specificity  Explained Variance |
| Hu^91^  2015  USA  D+IV+EV | 1 | R | Insured general population | Total Healthcare Costs  1) Outpatient, ED and inpatients days | D 848743 (848743)  IV 424371 (424371)  EV 1358153 (1358153) | Demographics  Prior utilization | 6 | Explained Variance |
| Huber^65^  2013  Switzerland  D+IV | 2 | R | Insured general population | Healthcare expenditure  1) Total annualized healthcare costs  a) Outpatient and  inpatient | D 436350 (436350; NA; NA)  V 10-fold cross-validation | Demographics  Enabling Family  Enabling Community  Evaluated Need | 12 | Explained Variance  Discrimination  Other (MAPE) |
| **Study**  First author  Year of publication  Country of publication  Development  Validation (Internal / External) | **Number of Unique Models** | **Pro- or Retrospective Data** | **Base Population Setting** | **Outcome**  Outcome Category  - Healthcare expenditure  - Healthcare utilization  - Clinical outcomes  Study definition of outcome | **Sample Size**  Development cohort  Validation cohort  (Participants with outcome) | **Type of Final Predictors**  - Demographics  - Social Structure  - Beliefs  - Enabling Family  - Enabling Community  - Perceived Need  - Evaluated Need  - Prior utilization | **Longest** **Prediction Timespan** | **Performance Measures**  - Sensitivity / specificity  - Explained variance  - Discrimination  - Calibration  - Classification  *-* Other |
| Huber^65^  2013  Switzerland  (continued) |  |  |  | b) Outpatient  c) Inpatient  Healthcare utilization  2) Outpatient visits  3) Any hospitalization  Clinical outcomes  4) Mortality |  |  |  |  |
| Izad Shenas^92^  2014  Canada  D+IV | 3 | R | Insured general population | Healthcare expenditure  1) Top 5% of total annualized healthcare expenditures  2) Top 10% of total annualized healthcare expenditures  3) Top 20% of total annualized healthcare expenditures | D 31704 (1585; 3170; 6341)  V 10-fold cross-validation | Demographics  Social Structure  Enabling Family  Enabling Community  Perceived Need  Evaluated Need  Prior utilization | 12 | Sensitivity / Specificity  Discrimination |
| Kharrazi^59^  2018  USA  D+IV | 12 | R | Insured general population | Healthcare expenditure  1) Annualized healthcare costs  a) Total  b) Pharmacy | D 59849 (59849; 2992; 1945; 5793)  V bootstrap of 300 runs | Demographics  Evaluated Need | 12 | Explained Variance  Discrimination  Other (MAPE) |
| **Study**  First author  Year of publication  Country of publication  Development  Validation (Internal / External) | **Number of Unique Models** | **Pro- or Retrospective Data** | **Base Population Setting** | **Outcome**  Outcome Category  - Healthcare expenditure  - Healthcare utilization  - Clinical outcomes  Study definition of outcome | **Sample Size**  Development cohort  Validation cohort  (Participants with outcome) | **Type of Final Predictors**  - Demographics  - Social Structure  - Beliefs  - Enabling Family  - Enabling Community  - Perceived Need  - Evaluated Need  - Prior utilization | **Longest** **Prediction Timespan** | **Performance Measures**  - Sensitivity / specificity  - Explained variance  - Discrimination  - Calibration  - Classification  *-* Other |
| Kharrazi^59^  2018  USA  (continued) |  |  |  | c) Medical  2) Top 5% of total annualized healthcare expenditures  Healthcare utilization  3) Any hospitalization  4) Any ED visit |  |  |  |  |
| Kim^93^  2019  South Korea  D+EV | 33 | R | Korean Population | Healthcare expenditure  1) Top 10% of total annualized healthcare expenditures | Total 354819 (35482)  D NA (NA)  V NA (NA) | Demographics  Enabling Family  Enabling Community  Perceived Need  Evaluated Need  Prior utilization | 12 | Discrimination  Other (Cost Capture) |
| Lauffenburger^66^  2017  USA  D+IV | 5 | R | Insured general population | Healthcare expenditure  1) High-Cost Trajectory  2) Top 5% of total annualized healthcare expenditures | D 499781 (156892 ; 24914)  V 499780 (157379; 25020 ) | Demographics  Evaluated Need  Prior utilization | 12 | Explained Variance  Discrimination  Classification |
| Lauffenburger^67^  2020  USA  D+IV | 12 | R | Medicare | Healthcare expenditure  1) Total monthly healthcare expenditure | D 164738 (164738) (16474)  V 164738 (164738) (16474) | Demographics  Social Structure  Enabling Family  Enabling Community  Prior utilization | 12 | Discrimination |
| **Study**  First author  Year of publication  Country of publication  Development  Validation (Internal / External) | **Number of Unique Models** | **Pro- or Retrospective Data** | **Base Population Setting** | **Outcome**  Outcome Category  - Healthcare expenditure  - Healthcare utilization  - Clinical outcomes  Study definition of outcome | **Sample Size**  Development cohort  Validation cohort  (Participants with outcome) | **Type of Final Predictors**  - Demographics  - Social Structure  - Beliefs  - Enabling Family  - Enabling Community  - Perceived Need  - Evaluated Need  - Prior utilization | **Longest** **Prediction Timespan** | **Performance Measures**  - Sensitivity / specificity  - Explained variance  - Discrimination  - Calibration  - Classification  *-* Other |
| Lauffenburger^67^  2020  USA  (continued) |  |  |  | 2) Top 10% of total annualized healthcare expenditures |  |  |  |  |
| Leininger^61^  2014  USA  D+IV | 7 | R | Medicaid | Healthcare utilization  **1)** ≥ 3 ED visits  2) ≥ 1 inpatient hospitalization  Healthcare expenditure  3) Top 10% of total annualized healthcare expenditures | D 34087 (3076; 3147; 3957) | Demographics  Social Structure  Enabling Family  Enabling Community  Perceived Need  Evaluated Need  Prior utilization | 12 | Sensitivity / Specificity  Discrimination |
| Li^94^  2019  Canada  V | 6 | P | Insured general population | Healthcare expenditure  1) Normalized cost risk scores | V 12,826,542 (13,293,352)  V 12,547,258 (12,991,652) | Demographics  Evaluated Need  Prior utilization | 12 | Explained Variance  Calibration  Other (MAPE) |
| Lu^95^  2015  USA  D | 8 | R | Enrollees in an indigent care program | Healthcare expenditure  1) Top 10% of total annualized healthcare expenditures | D 9624 (963) | Demographics  Social Structure  Evaluated Need  Prior utilization | 12 | Discrimination  Calibration |
| **Study**  First author  Year of publication  Country of publication  Development  Validation (Internal / External) | **Number of Unique Models** | **Pro- or Retrospective Data** | **Base Population Setting** | **Outcome**  Outcome Category  - Healthcare expenditure  - Healthcare utilization  - Clinical outcomes  Study definition of outcome | **Sample Size**  Development cohort  Validation cohort  (Participants with outcome) | **Type of Final Predictors**  - Demographics  - Social Structure  - Beliefs  - Enabling Family  - Enabling Community  - Perceived Need  - Evaluated Need  - Prior utilization | **Longest** **Prediction Timespan** | **Performance Measures**  - Sensitivity / specificity  - Explained variance  - Discrimination  - Calibration  - Classification  *-* Other |
| Lubanski^96^  2014  USA  V | 20 | R | Uninsured with ≥ 1 primary care visit at safety-net clinic | Healthcare expenditure  1) Top 10% of total annualized healthcare expenditures  Healthcare utilization  2) Any inpatient visit  3) Any Emergency Department (ED) visit  4) Any unnecessary ED visit (Emergency Severity Index triage level of 4 or 5) | D 4715 (472; 764; 2275; -) | Demographics  Evaluated Need  Prior utilization | 12 | Discrimination  Calibration |
| Maciejewski^97^  2005  USA  D | 15 | R | VA healthcare service | Healthcare expenditure  1) Total healthcare expenditure | D 14449 (14449) | Demographics Evaluted Need  Prior utilization | 12 | Explained Variance |
| Monterde^98^  2020  Spain  D | 4 | P | Insured general population | Healthcare utilization  1) Frequent attendance in primary care (≥12 visits)  2) Receiving of home care support  3) Receiving of social support visits | D 6102595 (860466; 292925; 164770; 945902) | Demographics  Enabling Family  Evaluated Need | 12 | Explained Variance  Discrimination |
| **Study**  First author  Year of publication  Country of publication  Development  Validation (Internal / External) | **Number of Unique Models** | **Pro- or Retrospective Data** | **Base Population Setting** | **Outcome**  Outcome Category  - Healthcare expenditure  - Healthcare utilization  - Clinical outcomes  Study definition of outcome | **Sample Size**  Development cohort  Validation cohort  (Participants with outcome) | **Type of Final Predictors**  - Demographics  - Social Structure  - Beliefs  - Enabling Family  - Enabling Community  - Perceived Need  - Evaluated Need  - Prior utilization | **Longest** **Prediction Timespan** | **Performance Measures**  - Sensitivity / specificity  - Explained variance  - Discrimination  - Calibration  - Classification  *-* Other |
| Monterde^98^  2020  Spain  (continued) |  |  |  | 4) Polypharmacy (≥8 drugs) |  |  |  |  |
| Ng^38^  2020  Singapore  D+IV+EV | 4 | R | Hospitalized population | Healthcare expenditure  1) Top 10% of total annualized healthcare expenditures for three consecutive years (Persistent High Utiliser)  2) Top 10% of total annualized healthcare expenditures for at least 1year, but less than three consecutive years (Transient High Utiliser) | D 67253 (5094; 62159)  IV repeated cross-validation  EV 19064 (1684; 17380) | Demographics  Social Structure  Evaluated Need  Prior utilization | 24 | Sensitivity / Specificity  Discrimination |
| Orueta^34^  2018  Spain  D+IV | 4 | R | Insured general population | Healthcare expenditure  1) Total annualized healthcare costs  2) Top 5% of total annualized healthcare expenditures | D 973400 (973400; 48670; 9734; 33869; 10053; 6890; 9155)  V 973484 (973484; 48734; 9897; 33884; 10288; 7072; 9392) | Demographics  Evaluated Need  Prior utilization | 12 | Explained Variance  Discrimination  Calibration |
| **Study**  First author  Year of publication  Country of publication  Development  Validation (Internal / External) | **Number of Unique Models** | **Pro- or Retrospective Data** | **Base Population Setting** | **Outcome**  Outcome Category  - Healthcare expenditure  - Healthcare utilization  - Clinical outcomes  Study definition of outcome | **Sample Size**  Development cohort  Validation cohort  (Participants with outcome) | **Type of Final Predictors**  - Demographics  - Social Structure  - Beliefs  - Enabling Family  - Enabling Community  - Perceived Need  - Evaluated Need  - Prior utilization | **Longest** **Prediction Timespan** | **Performance Measures**  - Sensitivity / specificity  - Explained variance  - Discrimination  - Calibration  - Classification  *-* Other |
| Orueta^34^  2018  Spain  (continued) |  |  |  | 3) Top 1% of total annualized healthcare expenditures  Healthcare utilization  4) ≥ 1 Emergency Hospitalization  5) Prolonged stay (sum of hospital bed days for causes other than obstetric and traumatic conditions >11 days)  6) Very prolonged stay (sum of hospital bed days for causes other than obstetric and traumatic conditions >15 days)  Clinical outcome  7) Mortality |  |  |  |  |
| Osawa^35^  2020  Japan  D+EV | 5 | R | Insured working population | Healthcare expenditure  1) Top 5% of total annualized healthcare expenditures | D 36316 (1816)  EV 36316 (1816) | Demographics  Perceived Need  Evaluated Need  Prior utilization | 12 | Sensitivity / Specificity  Discrimination  Calibration  Classification |
| **Study**  First author  Year of publication  Country of publication  Development  Validation (Internal / External) | **Number of Unique Models** | **Pro- or Retrospective Data** | **Base Population Setting** | **Outcome**  Outcome Category  - Healthcare expenditure  - Healthcare utilization  - Clinical outcomes  Study definition of outcome | **Sample Size**  Development cohort  Validation cohort  (Participants with outcome) | **Type of Final Predictors**  - Demographics  - Social Structure  - Beliefs  - Enabling Family  - Enabling Community  - Perceived Need  - Evaluated Need  - Prior utilization | **Longest** **Prediction Timespan** | **Performance Measures**  - Sensitivity / specificity  - Explained variance  - Discrimination  - Calibration  - Classification  *-* Other |
| Pacala^99^  2003  USA  D+IV | 3 | R | Medicare | Healthcare utilization  1) ≥ 2 hospitalizations in 1 year | D 6837 (462)  V 6845 (NA) | Demographics  Perceived Need  Evaluated Need  Prior utilization | 12 | Explained Variance  Calibration |
| Perkins^100^  2004  USA  D | 10 | P | Primary care >60 years | Healthcare expenditure  1) Total annualized healthcare expenditures  Clinical outcome  2) Mortality | D 3496 (3496; 98) | Demographics  Social Structure  Evaluated Need | 12 | Explained Variance  Discrimination |
| Pope^101^  1998  USA  D+EV | 9 | R | Medicare | Healthcare expenditure  1) Total annualized Medicare program expenditures | D 10893 (10893)  V 10532 (10532) | Demographics  Enabling Family  Perceived Need  Evaluated Need  Prior utilization | 12 | Explained Variance |
| Rakovski^31^  2002  USA  D+IV | 2 | R | VA healthcare service | Healthcare expenditure  1) Top 2% of total annualized healthcare expenditures | D 408599 (8172)  V 205278 (4106) | Demographics  Prior utilization | 12 | Sensitivity / Specificity  Discrimination |
| **Study**  First author  Year of publication  Country of publication  Development  Validation (Internal / External) | **Number of Unique Models** | **Pro- or Retrospective Data** | **Base Population Setting** | **Outcome**  Outcome Category  - Healthcare expenditure  - Healthcare utilization  - Clinical outcomes  Study definition of outcome | **Sample Size**  Development cohort  Validation cohort  (Participants with outcome) | **Type of Final Predictors**  - Demographics  - Social Structure  - Beliefs  - Enabling Family  - Enabling Community  - Perceived Need  - Evaluated Need  - Prior utilization | **Longest** **Prediction Timespan** | **Performance Measures**  - Sensitivity / specificity  - Explained variance  - Discrimination  - Calibration  - Classification  *-* Other |
| Reuben^60^  2002  USA  D+IV | 3 | R | People over 65 years of age | Healthcare utilization  1) ≥ 11 hospital days in 3 years | Total 5138 (1243)  D 2569 (NA)  V 2569 (NA) | Demographics  Social Structure  Perceived Need  Evaluated Need  Prior utilization | 36 | Sensitivity / Specificity  Explained Variance  Discrimination |
| Robst^102^  2015  USA  D | 4 | R | Medicaid | Healthcare expenditure  1) Top 1% of total annualized healthcare expenditures in the following year  2) Top 1% of total annualized healthcare expenditures in the following year given persistent top-1% use for five years prior  3) Persistent top 1% of total annualized healthcare expenditures in the following five years given top-1% use in the year prior  4) Persistent top 1% of total annualized healthcare expenditures in the | D 512.631 (7432; 3325; 6367; 4057) | Demographics  Social Structure  Enabling Family  Perceived Need  Prior utilization | 60 | Sensitivity / Specificity  Calibration |
| **Study**  First author  Year of publication  Country of publication  Development  Validation (Internal / External) | **Number of Unique Models** | **Pro- or Retrospective Data** | **Base Population Setting** | **Outcome**  Outcome Category  - Healthcare expenditure  - Healthcare utilization  - Clinical outcomes  Study definition of outcome | **Sample Size**  Development cohort  Validation cohort  (Participants with outcome) | **Type of Final Predictors**  - Demographics  - Social Structure  - Beliefs  - Enabling Family  - Enabling Community  - Perceived Need  - Evaluated Need  - Prior utilization | **Longest** **Prediction Timespan** | **Performance Measures**  - Sensitivity / specificity  - Explained variance  - Discrimination  - Calibration  - Classification  *-* Other |
| Robst^102^  2015  USA  (continued) |  |  |  | following three years given persistent top-1% use for three years prior |  |  |  |  |
| Rosella^37^  2018  Canada  D+EV | 1 | P | Insured general population | Healthcare expenditure  1) Top 5% of total annualized healthcare expenditures | D 3502 (175)  V 1611 (81) | Demographics  Social Structure  Enabling Family  Perceived Need  Evaluated Need | 60 | Explained Variance  Discrimination  Calibration |
| Rosella^39^  2020  Canada  V | 1 | P | Insured general population | Healthcare expenditure  1) Top 5% of total annualized healthcare expenditures | V 10504 (1145) | Demographics  Social Structure  Enabling Family  Perceived Need  Evaluated Need | 60 | Explained Variance  Discrimination  Calibration |
| Schiltz^103^  2017  USA  D+IV+EV | 2 | R | Medicare | Healthcare expenditure  1) Top 25% of total healthcare expenditures (PMPM Medicare amount reimbursed) | D 5771 (1443; 2366)  IV 10-fold cross-validation  EV 5186 (1297; 1739) | Demographics  Perceived Need  Evaluated Need | 12 | Discrimination |
| **Study**  First author  Year of publication  Country of publication  Development  Validation (Internal / External) | **Number of Unique Models** | **Pro- or Retrospective Data** | **Base Population Setting** | **Outcome**  Outcome Category  - Healthcare expenditure  - Healthcare utilization  - Clinical outcomes  Study definition of outcome | **Sample Size**  Development cohort  Validation cohort  (Participants with outcome) | **Type of Final Predictors**  - Demographics  - Social Structure  - Beliefs  - Enabling Family  - Enabling Community  - Perceived Need  - Evaluated Need  - Prior utilization | **Longest** **Prediction Timespan** | **Performance Measures**  - Sensitivity / specificity  - Explained variance  - Discrimination  - Calibration  - Classification  *-* Other |
| Schiltz^103^  2017  USA  (continued) |  |  |  | Healthcare utilization  2) Inpatient utilization (≥ 1 claim for a hospital stay in two years) |  |  |  |  |
| Sheets^104^  2016  USA  D | 1 | R | Primary care | Healthcare expenditure  1) Total annualized healthcare expenditures  Healthcare utilization  2) Emergency hospitalizations  3) Observation hospitalizations  4) Inpatient hospitalizations | D 9581 (9581; 1101; 1574; 1972) | Evaluated Need  Prior utilization | 12 | NA |
| Sheets^105^  2017  USA  D+IV | 1 | R | Primary care | Healthcare expenditure  1) Top 5% of total annualized healthcare expenditures | D 9568 (478)  V 9568 (478) | Demographics  Social Structure  Enabling Family  Evaluated Need  Prior utilization | 12 | Sensitivity / Specificity  Discrimination |
| Snider^106^  2014  USA  D+IV | 1 | R | Insured general population | Healthcare expenditure  1) Top 20% of total annualized healthcare expenditures | D 105181 (21036)  V 11687 (2337) | Demographics  Evaluated Need  Prior utilization | 12 | Other (MERS score) |
| **Study**  First author  Year of publication  Country of publication  Development  Validation (Internal / External) | **Number of Unique Models** | **Pro- or Retrospective Data** | **Base Population Setting** | **Outcome**  Outcome Category  - Healthcare expenditure  - Healthcare utilization  - Clinical outcomes  Study definition of outcome | **Sample Size**  Development cohort  Validation cohort  (Participants with outcome) | **Type of Final Predictors**  - Demographics  - Social Structure  - Beliefs  - Enabling Family  - Enabling Community  - Perceived Need  - Evaluated Need  - Prior utilization | **Longest** **Prediction Timespan** | **Performance Measures**  - Sensitivity / specificity  - Explained variance  - Discrimination  - Calibration  - Classification  *-* Other |
| Tamang^46^  2017  Denmark  D+EV | 6 | P | Insured general population | Healthcare expenditure  1) Top-10% of total annualized healthcare expenditures in whole population  2) Top-10% of total annualized healthcare expenditures for non-high cost population | D1 NA (NA)  D2 NA (NA)  EV1 1557950 (155795)  EV2 1402155 (140216) | Demographics  Social Structure  Enabling Community  Prior utilization | 12 | Discrimination  Other (cost capture) |
| Weinberger^107^  1992  USA  D+IV | 2 | P | Patients discharged from General Medicine | Healthcare expenditure  1) Log transformed total costs  2) Log transformed nonelective readmission costs | D 315 (315)  V 157 (157) | Demographics  Evaluated Need  Prior utilization | 6 | Explained Variance  Calibration |
| Weir^108^  2020  Canada  V | 3 | P | Insured general population | Healthcare expenditure  1) Top 1% of total annualized healthcare expenditures  2) Top 5% of total annualized healthcare expenditures  3) Top 10% of total annualized healthcare expenditures | V 11684427 (1168441; 584222; 116845) | Demographics  Evaluated Need | 12 | Sensitivity / Specificity  Calibration |
| **Study**  First author  Year of publication  Country of publication  Development  Validation (Internal / External) | **Number of Unique Models** | **Pro- or Retrospective Data** | **Base Population Setting** | **Outcome**  Outcome Category  - Healthcare expenditure  - Healthcare utilization  - Clinical outcomes  Study definition of outcome | **Sample Size**  Development cohort  Validation cohort  (Participants with outcome) | **Type of Final Predictors**  - Demographics  - Social Structure  - Beliefs  - Enabling Family  - Enabling Community  - Perceived Need  - Evaluated Need  - Prior utilization | **Longest** **Prediction Timespan** | **Performance Measures**  - Sensitivity / specificity  - Explained variance  - Discrimination  - Calibration  - Classification  *-* Other |
| Wells^109^  2016  USA  D+IV+EV | 2 | P | Insured general population | Healthcare expenditure  1) Member-level annual health care expenditures after assignment to cohort based on equal proportions of current cost (low, medium, high, very high)  2) Top 10% of total annualized healthcare expenditures | D 105324 (51239; 54085)  V 27043 (NA) | Demographics  Social Structure  Evaluated Need  Prior utilization | 12 | Sensitivity / Specificity  Other (Cost Capture) |
| Wherry^33^  2014  USA  D+IV | 11 | R | Medicaid | Healthcare utilization  1) ≥ 1 Hospitalization  2) ≥ 2 ED visits  Healthcare expenditure  3) Top 10% of total annualized healthcare expenditures | Total 6725 (637; 483; 642)  D 3363 (NA)  V 3362 (NA) | Demographics  Social Structure  Enabling Family  Enabling Community  Perceived Need  Evaluated Need  Prior utilization | 12 | Sensitivity / Specificity  Discrimination  Calibration |
| Yang^110^  2018  USA  D+IV | 4 | R | Medicaid | Healthcare expenditure  1) Top 10% of total annualized healthcare expenditures | D 1.734.896 (173.490)  V 1.734.896 (173.490) | Demographics  Social Structure  Perceived Need  Evaluated Need | 12 | Explained Variance  Other (RMSEA) |
| **Study**  First author  Year of publication  Country of publication  Development  Validation (Internal / External) | **Number of Unique Models** | **Pro- or Retrospective Data** | **Base Population Setting** | **Outcome**  Outcome Category  - Healthcare expenditure  - Healthcare utilization  - Clinical outcomes  Study definition of outcome | **Sample Size**  Development cohort  Validation cohort  (Participants with outcome) | **Type of Final Predictors**  - Demographics  - Social Structure  - Beliefs  - Enabling Family  - Enabling Community  - Perceived Need  - Evaluated Need  - Prior utilization | **Longest** **Prediction Timespan** | **Performance Measures**  - Sensitivity / specificity  - Explained variance  - Discrimination  - Calibration  - Classification  *-* Other |
| Yen^111^  2003  USA  D+IV | 3 | P | Insured general population | Healthcare expenditure  1) Total annualized medical expenditures  2) Total annualized health services expenditures  3) Total annualized drug expenditures | D 10172 (10172)  V 9689 (9689) | Demographics  Perceived Need  Evaluated Need | 12 | Explained Variance  Calibration |
| Zhang^112^  2020  Canada  D+IV | 3 | P | Primary care | Healthcare expenditure  1) Top 5% of total annualized healthcare expenditures | D 221738 (11087)  V 55435 (2772) | Demographics  Social Structure  Enabling Family  Perceived Need  Evaluated Need  Prior utilization | 12 | Discrimination |
| Ziring^113^  2018  USA | 1 | P | Safety Net System Patients | Healthcare utilization  1) ≥ 10 days in inpatient care or the emergency room | D 643475 (18661)  V 160868 (NA) | Demographics  Social Structure  Enabling Family  Evaluated Need  Prior utilization | 12 | Sensitivity / Specificity  Discrimination |

USA = United States of America; UK = United Kingdom; ROB = risk of bias; BIC = Bayesian Information Criterion; MAPE = mean absolute percentage error; MERS = medical expenditure risk

Score; RMSEA = root mean square error of approximation

D indicates development study; IV indicates internal validation; EV indicates external validation; R indicates retrospective; P indicates prospective; NA indicates not available in spite of inquiry to the author.

**eTable 2. Prediction model Risk Of Bias Assessment Tool (PROBAST) results on Risk of Bias, concern for applicability and overall judgement**

|  |  | **Risk of Bias** | | | |  | **Applicability** | | |  | **Overall** | |
| --- | --- | --- | --- | --- | --- | --- | --- | --- | --- | --- | --- | --- |
|  |  | **Participants** | **Predictors** | **Outcome** | **Analysis** |  | **Participants** | **Predictors** | **Outcome** |  | **ROB** | **Applicability** |
| AlSnih^75^  2006  USA |  | + | - | - | + |  | ? | - | - |  | + | ? |
| Anderson^76^  2004  USA |  | ? | - | ? | + |  | ? | - | - |  | + | ? |
| Bertakis^77^  2010  USA |  | + | - | ? | + |  | ? | - | ? |  | + | ? |
| Billings^78^  2006  UK |  | ? | ? | - | + |  | ? | ? | ? |  | + | ? |
| Blumenthal^79^  2017  USA |  | - | - | - | + |  | - | - | - |  | + | - |
| Bosworth^80^  2000  USA |  | ? | - | - | + |  | - | - | - |  | + | - |
| Boult^81^  2004  USA |  | - | - | ? | + |  | - | - | - |  | + | - |
| Chang^82^  2002  Taiwan |  | - | - | - | + |  | - | - | - |  | + | - |
| Charlson^83^  2014  USA |  | - | - | - | ? |  | - | - | - |  | - | - |
| Charlson^84^  2008  USA |  | ? | - | - | + |  | ? | - | - |  | + | ? |
|  |  | **Risk of Bias** | | | |  | **Applicability** | | |  | **Overall** | |
|  |  | **Participants** | **Predictors** | **Outcome** | **Analysis** |  | **Participants** | **Predictors** | **Outcome** |  | **ROB** | **Applicability** |
| Chechulin^36^  2014  Canada |  | - | - | - | - |  | - | - | - |  | - | - |
| Cohen^85^  2006  USA |  | ? | ? | - | ? |  | - | - | - |  | ? | - |
| Cunningham^86^  2017  USA |  | - | - | - | ? |  | - | - | - |  | ? | - |
| DeSalvo^57^  2005  USA |  | - | - | - | + |  | - | - | - |  | + | - |
| DeSalvo^63^  2009  USA |  | ? | - | ? | ? |  | ? | - | + |  | ? | + |
| Dove^87^  2003  USA |  | ? | - | - | + |  | - | - | - |  | + | - |
| Fleishman^88^  2006  USA |  | ? | - | ? | + |  | ? | - | + |  | + | + |
| Fleishman^62^  2010  USA |  | - | - | - | + |  | - | - | - |  | + | - |
| Freedman^56^  1996  USA |  | - | - | - | + |  | - | - | - |  | + | - |
| Frost^54^  2017  Canada |  | - | - | - | ? |  | - | - | - |  | ? | - |
| Haas^32^  2013  USA |  | - | - | - | - |  | - | - | - |  | - | - |
|  |  | **Risk of Bias** | | | |  | **Applicability** | | |  | **Overall** | |
|  |  | **Participants** | **Predictors** | **Outcome** | **Analysis** |  | **Participants** | **Predictors** | **Outcome** |  | **ROB** | **Applicability** |
| Harrison^89^  2012  USA |  | ? | ? | - | + |  | - | - | - |  | + | - |
| Heins^90^  2020  The Netherlands |  | - | - | - | + |  | - | - | - |  | + | - |
| Hu^91^  2015  US |  | - | - | - | + |  | - | - | - |  | + | - |
| Huber^65^  2013  Switzerland |  | ? | - | - | ? |  | ? | - | - |  | ? | ? |
| Izad Shenas^92^  2014  Canada |  | ? | - | ? | + |  | ? | - | - |  | + | ? |
| Kharrazi^59^  2018  USA |  | - | - | - | + |  | - | - | - |  | + | - |
| Kim^93^  2019  South Korea |  | ? | - | ? | ? |  | ? | - | - |  | ? | ? |
| Lauffenburger^66^  2017  USA |  | - | - | - | ? |  | - | ? | - |  | ? | ? |
| Lauffenburger^67^  2020  USA |  | - | - | - | + |  | - | - | - |  | + | - |
| Leininger^61^  2014  USA |  | + | ? | ? | + |  | ? | - | - |  | + | ? |
| Li^94^  2019  Canada |  | - | - | - | - |  | - | - | - |  | - | - |
|  |  | **Risk of Bias** | | | |  | **Applicability** | | |  | **Overall** | |
|  |  | **Participants** | **Predictors** | **Outcome** | **Analysis** |  | **Participants** | **Predictors** | **Outcome** |  | **ROB** | **Applicability** |
| Lu^95^  2015  USA |  | - | - | - | + |  | - | - | - |  | + | - |
| Lubanski^96^  2014  USA |  | - | - | - | + |  | - | - | - |  | + | - |
| Maciejewski^97^  2005  USA |  | - | - | - | + |  | - | - | - |  | + | - |
| Monterde^98^  2020  Spain |  | - | - | - | + |  | - | - | - |  | + | - |
| Ng^38^  2020  Singapore |  | - | - | - | ? |  | - | - | - |  | ? | - |
| Orueta^34^  2018  Spain |  | - | ? | - | ? |  | - | - | - |  | ? | - |
| Osawa^35^  2020  Japan |  | - | - | - | - |  | - | - | - |  | - | - |
| Pacala^99^  2003  USA |  | + | - | - | + |  | - | - | - |  | + | - |
| Perkins^100^  2004  USA |  | + | - | - | + |  | - | - | - |  | + | - |
| Pope^101^  1998  USA |  | - | - | - | + |  | - | - | - |  | + | - |
| Rakovski^31^  2002  USA |  | - | - | - | ? |  | - | - | - |  | ? | - |
|  |  | **Risk of Bias** | | | |  | **Applicability** | | |  | **Overall** | |
|  |  | **Participants** | **Predictors** | **Outcome** | **Analysis** |  | **Participants** | **Predictors** | **Outcome** |  | **ROB** | **Applicability** |
| Reuben^60^  2002  USA |  | + | - | + | + |  | ? | - | - |  | + | - |
| Robst^102^  2015  USA |  | - | - | ? | + |  | ? | - | - |  | + | - |
| Rosella^37^  2018  Canada |  | - | - | - | ? |  | - | - | - |  | ? | - |
| Rosella^39^  2020  Canada |  | - | - | - | - |  | - | - | - |  | - | - |
| Schiltz^103^  2017  USA |  | - | ? | ? | + |  | - | - | - |  | + | - |
| Sheets^104^  2016  USA |  | ? | ? | - | + |  | ? | - | + |  | + | + |
| Sheets^105^  2017  USA |  | ? | - | - | + |  | ? | - | ? |  | + | ? |
| Snider^106^  2014  USA |  | - | - | ? | + |  | - | - | ? |  | + | ? |
| Tamang^46^  2017  Denmark |  | - | - | - | + |  | - | - | - |  | + | - |
| Weinberger^107^  1992  USA |  | ? | ? | + | + |  | ? | ? | ? |  | + | ? |
| Weir^108^  2020  Canada |  | - | ? | - | ? |  | - | ? | - |  | ? | ? |
|  |  | **Risk of Bias** | | | |  | **Applicability** | | |  | **Overall** | |
|  |  | **Participants** | **Predictors** | **Outcome** | **Analysis** |  | **Participants** | **Predictors** | **Outcome** |  | **ROB** | **Applicability** |
| Wells^109^  2016  USA |  | + | ? | ? | ? |  | ? | ? | ? |  | + | ? |
| Wherry^33^  2014  USA |  | - | - | - | + |  | - | - | - |  | + | - |
| Yang^110^  2018  USA |  | - | ? | + | ? |  | - | ? | + |  | + | + |
| Yen^111^  2003  USA |  | - | - | - | + |  | - | - | - |  | + | - |
| Zhang^112^  2020 |  | - | - | + | + |  | - | - | ? |  | + | ? |
| Ziring^113^  2018  USA |  | - | - | - | - |  | - | - | - |  | - | - |
| Wells^109^  2016  USA |  | - | - | - | ? |  | - | - | - |  | ? | - |

PROBAST = Prediction model Risk Of Bias Assessment Tool; ROB = risk of bias. USA = United States of America

* + indicates high ROB/ high concern regarding applicability; − indicates low ROB/ low concern regarding applicability; and ? indicates unclear ROB/unclear concern regarding applicability.

**eTable 3. Summary of findings on contacting authors of eligible studies**

| **Study**  First author  Year of Publication  Country | Did the eligible study  have valid contact data? | Did you contact one or more  authors of the eligible study? | Did the authors of the  eligible study reply? | Did you obtain all the  information requested  from the eligible study? | What information was and  was not obtained from the  eligible study?  - sample size  - number of events  - handling of missing data  - coefficients and intercepts  - performance measures  - validation procedures | How was the obtained  information used? | What were the consequences of the obtained information for the systematic review? | Was a draft of the systematic  review sent to the original  investigators before the  publiction of the systematic  review? | Did the contacted author approve the correctness and accuracy of the use of the obtained information in the systematic review? |
| --- | --- | --- | --- | --- | --- | --- | --- | --- | --- |
| Anderson  2004  USA | Yes | Yes | No | No | sample size  number of events  performance measures | NA | Calculation of enrolees and events based on percentages rather thn original numbers | No | No |
| Bertakis^77^  2010  USA | Yes | Yes | Yes | No | handling of missing data  coefficients and intercepts  perforance measures | NA | No additional information was included in review | No | No |
| Billings^78^  2006  UK | Yes | Yes | No | No | sample size  number of events | A | No additional information was included in review | No | No |
| Blumenthal^79^  2017  USA | Yes | Yes | No | No | number of events  handlin of missing data  performance measures | NA | Calculation of number of events based on percentages rather than original numbers | No | No |
| Bosworth  2000  USA | Yes | Yes | No | No | sample size  number of events | NA | No additional information was included in review | No | No |
| **Study**  First author  Year of Publication  Country | Did the eligible study  have valid contact data? | Did you contact one or more  authors of the eligible study? | Did the authors of the  eligible study reply? | Did you obtain all the  information requested  from the eligible study? | What information was and  was not obtained from the  eligible study?  - sample size  - number of events  - handling of missing data  - coefficients and intercepts  - performance measures  - validation procedures | How was the obtained  information used? | What were the consequences  of the obtained information for  the systematic review? | Was a draft of the systematic  review sent to the original  investigators before the  publication of the systematic  review? | Did the contacted author  approve the correctness and  accuracy of the use of the  obtained information in the  systematic review? |
| Huber^65^  2013  Switzerland | Yes | Yes | No | No | sample size  number of events | NA | No additional information was included in review | No | No |
| Kim^93^  2019  South Korea | Yes | Yes | No | No | sample size  handling of missing data | NA | No additional information was included in review | No | No |
| Lauffenburger^66^  2017  USA | Yes | Yes | Yes | Yes | number of events  handling of missing data | Completion of eTable 1 and complementary to PROBAST | Additional information was included in review | No | No |
| Osawa^35^  2020  Japan | Yes | Yes | No | No | sample size  number of events | NA | No additional information was included in review | No | No |
| Pacala^99^  2003  USA | Yes | Yes | No | No | sample size  number of events | NA | No additional information was included in review | No | No |
| Reuben^60^  2002  USA | Yes | Yes | No | No | sample size  number of events | NA | No additional information was included in review | No | No |
| Schiltz^103^  2017  USA | Yes | Yes | Yes | Yes | sample size  number of events  handling of missing data | Completion of eTable 1 and complementary to PROBAST | Additional information was included in review | No | No |
| **Study**  First author  Year of Publication  Country | Did the eligible study  have valid contact data? | Did you contact one or more  authors of the eligible study? | Did the authors of the  eligible study reply? | Did you obtain all the  information requested  from the eligible study? | What information was and  was not obtained from the  eligible study?  - sample size  - number of events  - handling of missing data  - coefficients and intercepts  - performance measures  - validation procedures | How was the obtained  information used? | What were the consequences  of the obtained information for  the systematic review? | Was a draft of the systematic  review sent to the original  investigators before the  publication of the systematic  review? | Did the contacted author  approve the correctness and  accuracy of the use of the  obtained information in the  systematic review? |
| Wells^109^  2016  USA | No | Yes | No | No | sample size  number of events  validation procedures | NA | No additional information was included in review | No | No |
| Wherry^33^  2014  USA | Yes | Yes | No | No | number of events | NA | No additional information was included in review | No | No |
| Ziring^113^  2018  USA | Yes | Yes | No | No | sample size  number of events  handling of missing data  coefficients and intercepts | NA | No additional information was included in review | No | No |

USA = United States of America; UK = United Kingdom; NA = Not applicable; PROBAST = Prediction model Risk Of Bias Assessment Tool

* Authors were contacted when there were missing data or unclarities regarding the study population, predicted outcome, sample size, number of candidate predictors, final predictors, prediction timespan and/or performance measures.^19^

# eReferences

References additional to the main manuscript for the benefit of the supplemental material.

56. Freedman JD, Beck A, Robertson B, Calonge BN, Gade G. Using a mailed survey to predict hospital admission among patients older than 80*. J Am Geriatr Soc*. 1996;44(6):689-692.

57. DeSalvo KB, Fan VS, McDonell MB, Fihn SD. Predicting mortality and healthcare utilization with a single question*. Health Serv Res*. 2005;40(4):1234-1246.

58. Charlson M, Wells MT, Ullman R, King F, Shmukler C . The Charlson comorbidity index can be used prospectively to identify patients who will incur high future costs*. PloS one*. 2014;9(12):e112479.

59. Kharrazi H, Chang H, Heins S, Weiner JP, Gudzune KA. Assessing the Impact of Body Mass Index Information on the Performance of Risk Adjustment Models in Predicting Healthcare Costs and Utilization*. Med Care*. 2018;56(12):1042.

60. Reuben DB, Keeler E, Seeman TE, Sewall A, Hirsch SH, Guralnik JM. Development of a method to identify seniors at high risk for high hospital utilization*. Med Care*. 2002:782-793.

61. Leininger LJ, Friedsam D, Voskuil K, DeLeire T. Predicting high-need cases among new Medicaid enrollees*. Am J Manag Care*. 2014;20(9):e399-e407.

62. Fleishman JA, Cohen JW. Using information on clinical conditions to predict high-cost patients*. Health Serv Res*. 2010;45(2):532-552. https://doi.org/10.1111/j.1475-6773.2009.01080.x.

63. DeSalvo KB, Jones TM, Peabody J, et al. Health care expenditure prediction with a single item, self-rated health measure*. Med Care*. 2009:440-447.

64. Kim YJ, Park H. Improving prediction of high-cost health care users with medical check-up data *. Big data*. 2019;7(3):163-175.

65. Huber CA, Schneeweiss S, Signorell A, Reich O. Improved prediction of medical expenditures and health care utilization using an updated chronic disease score and claims data*. J Clin Epidemiol*. 2013;66(10):1118-1127.

66. Lauffenburger JC, Franklin JM, Krumme AA, et al. Longitudinal patterns of spending enhance the ability to predict costly patients*. Med Care*. 017;55(1):64-73.

67. Lauffenburger JC, Mahesri M, Choudhry NK. Use of Data-Driven Methods to Predict Long-term Patterns of Health Care Spending for Medicare Patients*. JAMA network open*. 2020;3(10):e2020291.

68. Covidence systematic review software, Veritas Health Innovation, Melbourne, Australia.

69. Kansagara D, Englander H, Salanitro A, et al. Risk prediction models for hospital readmission: a systematic review*. JAMA*. 2011;306(15):1688-1698. doi: 10.1001/jama.2011.1515 [doi].

70. Zhou H, Della PR, Roberts P, Goh L, Dhaliwal SS. Utility of models to predict 28-day or 30-day unplanned hospital readmissions: an updated systematic review*. BMJ open*. 2016;6(6):e011060.

71. Moons KG, de Groot JA, Bouwmeester W, et al. Critical appraisal and data extraction for systematic reviews of prediction modelling studies: the CHARMS checklist*. PLoS Med*. 2014;11(10):e1001744.

72. Collins GS, Reitsma JB, Altman DG, Moons KG. Transparent reporting of a multivariable prediction model for individual prognosis or diagnosis (TRIPOD) the TRIPOD statement*. Circulation*. 2015;131(2):211-219.

73. Riley RD, Snell KI, Ensor J, et al. Minimum sample size for developing a multivariable prediction model: Part I–Continuous outcomes*. Stat Med*. 2019;38(7):1262-1275.

74. Riley RD, Snell KI, Ensor J, et al. Minimum sample size for developing a multivariable prediction model: PART II‐binary and time‐to‐event outcomes*. Stat Med*. 2019;38(7):1276-1296.

75. Al Snih S, Markides KS, Ray LA, Freeman JL, Ostir GV, Goodwin JS. Predictors of healthcare utilization among older Mexican Americans. *Ethn Dis*. 2006;16(3):640-646.

76. Anderson RT, Balkrishnan R, Camacho F. Risk classification of Medicare HMO enrollee cost levels using a decision-tree approach*. Am J Manag Care*. 2004;10(2; PART 1):89-98.

77. Bertakis KD, Azari R. Patient gender differences in the prediction of medical expenditures*. Journal of Women's Health*. 2010;19(10):1925-1932.

78. Billings J, Dixon J, Mijanovich T, Wennberg D. Case finding for patients at risk of readmission to hospital: development of algorithm to identify high risk patients*. BMJ*. 2006;333(7563):327.

79. Blumenthal KJ, Chang Y, Ferris TG, et al. Using a self-reported global health measure to identify patients at high risk for future healthcare utilization*. Journal of general internal medicine*. 2017;32(8):877-882.

80. Bosworth HB, Butterfield MI, Stechuchak KM, Bastian LA. The relationship between self-rated health and health care service use among women veterans in a primary care clinic*. Womens Health Issues*. 2000;10(5):278-285.

81. Boult C, Kessler J, Urdangarin C, Boult L, Yedidia P. Identifying workers at risk for high health care expenditures: a short questionnaire*. Disease Management*. 2004;7(2):124-135.

82. Chang R, Lin W, Hsieh C, Chiang T. Healthcare utilization patterns and risk adjustment under Taiwan's National Health Insurance system. *J Formosan Med Assoc*. 2002;101(1):52-59.

83. Charlson M, Wells MT, Ullman R, King F, Shmukler C. The Charlson comorbidity index can be used prospectively to identify patients who will incur high future costs*. PloS one*. 2014;9(12):e112479.

84. Charlson ME, Charlson RE, Peterson JC, Marinopoulos SS, Briggs WM, Hollenberg JP. The Charlson comorbidity index is adapted to predict costs of chronic disease in primary care patients*. J Clin Epidemiol*. 2008;61(12):1234-1240.

85. Cohen SB, Ezzati-Rice T, Yu W. The utility of extended longitudinal profiles in predicting future health care expenditures*. Med Care*. 2006:I45-I53.

86. Cunningham PJ. Predicting high-cost privately insured patients based on self-reported health and utilization data*. Am J Manag Care*. 2017;23(7):215-222.

87. Dove HG, Duncan I, Robb A. A prediction model for targeting low-cost, high-risk members of managed care organizations*. Am J Manag Care*. 2003;9(5):381-389.

88. Fleishman JA, Cohen JW, Manning WG, Kosinski M. Using the SF-12 health status measure to improve predictions of medical expenditures*. Med Care*. 2006:I54-I63.

89. Harrison PL, Pope JE, Coberley CR, Rula EY. Evaluation of the relationship between individual well-being and future health care utilization and cost*. Population health management*. 2012;15(6):325-330.

90. Heins M, Korevaar J, Schellevis F, Rijken M. Identifying multimorbid patients with high care needs-A study based on electronic medical record data*. European Journal of General Practice*. 2020;26(1):189-195.

91. Hu Z, Hao S, Jin B, et al. Online prediction of health care utilization in the next six months based on electronic health record information: a cohort and validation study*. Journal of medical Internet research*. 2015;17(9):e219.

92. Shenas SAI, Raahemi B, Tekieh MH, Kuziemsky C. Identifying high-cost patients using data mining techniques and a small set of non-trivial attributes*. Comput Biol Med*. 2014;53:9-18.

93. Kim YJ, Park H. Improving prediction of high-cost health care users with medical check-up data*. Big data*. 2019;7(3):163-175.

94. Li Y, Weir S, Steffler M, Shaikh S, Wright JG, Kantarevic J. Using diagnoses to estimate health care cost risk in Canada*. Med Care*. 2019;57(11):875.

95. Lu J, Britton E, Ferrance J, Rice E, Kuzel A, Dow A. Identifying Future High Cost Individuals within an Intermediate Cost Population*. Quality in primary care*. 2015;23(6):318.

96. Lubanski E, Rozario N, Moore CG, et al. Traditional Risk Indices as Predictors of Future Utilization and Charges in the Context of Population Health for an Uninsured Cohort*. eGEMs*. 2017;5(1).

97. Maciejewski ML, Liu C, Derleth A, McDonell M, Anderson S, Fihn SD. The performance of administrative and self‐reported measures for risk adjustment of Veterans Affairs expenditures*. Health Serv Res*. 2005;40(3):887-904.

98. Monterde D, Vela E, Clèries M, Garcia-Eroles L, Roca J, Pérez-Sust P. Multimorbidity as a predictor of health service utilization in primary care: a registry-based study of the Catalan population*. BMC family practice*. 2020;21(1):1-9.

99. Pacala JT, Boult C, Urdangarin C, McCaffrey D. Using self‐reported data to predict expenditures for the health care of older people*. J Am Geriatr Soc*. 2003;51(5):609-614.

100. Perkins AJ, Kroenke K, Unützer J, et al. Common comorbidity scales were similar in their ability to predict health care costs and mortality*. J Clin Epidemiol*. 2004;57(10):1040-1048.

101. Pope GC, Adamache KW, Walsh EG, Khandker RK. Evaluating alternative risk adjusters for Medicare*. Health Care Financ Rev*. 1998;20(2):109.

102. Robst J. Developing models to predict persistent high-cost cases in Florida Medicaid*. Population health management*. 2015;18(6):467-476.

103. Schiltz NK, Warner DF, Sun J, et al. Identifying specific combinations of multimorbidity that contribute to health care resource utilization: an analytic approach*. Med Care*. 2017;55(3):276.

104. Sheets L, Popejoy L, GCNS-BC APRN MK, Petroski G, Parker JC. Identifying patients at risk of high healthcare utilization. 2016;2016:1129.

105. Sheets L, Petroski GF, Zhuang Y, et al. Combining Contrast Mining with Logistic Regression To Predict Healthcare Utilization in a Managed Care Population*. Appl Clin Inform*. 2017;8(2):430-446. doi: 10.4338/ACI-2016-05-RA-0078 [doi].

106. Snider JT, Bognar K, Globe D, et al. Identifying patients at risk for high medical costs and good candidates for obesity intervention*. American Journal of Health Promotion*. 2014;28(4):218-227.

107. Weinberger M, Smith DM, Katz BP, Moore PS, Kalasinski LA. Predicting post-hospital discharge health care costs. *J Ambulatory Care Manage*. 1992;15(1):29-37.

108. Weir S, Steffler M, Li Y, Shaikh S, Wright JG, Kantarevic J. Use of the Population Grouping Methodology of the Canadian Institute for Health Information to predict high-cost health system users in Ontario*. CMAJ*. 2020;192(32):E907-E912.

109. Wells AR, Guo X, Coberley CR, Pope JE. Integrating Well-Being Information and the Multidimensional Adaptive Prediction Process to Estimate Individual-Level Future Health Care Expenditure Levels*. Population health management*. 2016;19(6):429-438.

110. Yang C, Delcher C, Shenkman E, Ranka S. Machine learning approaches for predicting high cost high need patient expenditures in health care*. Biomedical engineering online*. 2018;17(1):131.

111. Yen L, McDonald T, Hirschland D, Edington DW. Association between wellness score from a health risk appraisal and prospective medical claims costs*. Journal of occupational and environmental medicine*. 2003;45(10):1049-1057.

112. Zhang Y, Khullar D, Wu Y, Casalino LP, Kaushal R. Identifying Patients with Persistent Preventable Utilization Offers an Opportunity to Reduce Unnecessary Spending*. Journal of General Internal Medicine*. 2020;35(12):3534-3541.

113. Ziring J, Gogia S, Newton-Dame R, Singer J, Chokshi DA. An All-Payer Risk Model for Super-Utilization in a Large Safety Net System*. Journal of general internal medicine*. 2018;33(5):596-598.
