# Supplementary material for: Prediction Models for Future High-Need High-Cost Healthcare Use: a Systematic Review
Source: J Gen Intern Med. 2022 Jan 11;37(7):1763–70. doi: 10.1007/s11606-021-07333-z (PMC9130365; doi:10.1007/s11606-021-07333-z)
Supplement: Supplementary file 2 — (DOCX 19 kb) [file 11606_2021_7333_MOESM2_ESM.docx]

| **Model** | **Study Population** | **Outcome** | **Prediction**  **Timespan, months** | **Natural log EPV** | **C-statistic** | **ROB** |
| --- | --- | --- | --- | --- | --- | --- |
| A^56^ | General population | U \| Any acute care visit | 4.5 | 2,9 | 0,63 | high |
| B^57^ | Veterans Affairs healthcare service | U \| Top 10% visits | 12 | 3,9 | 0,64 | high |
| C^58^ | General population | C \| Top-10% cost-distribution | 12 | 7,5 | 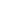0,65 | high |
| D^59^ | General population | C \| Top 5% cost-distribution | 12 | 5,8 | 0,67 | high |
| E^60^ | Other | U \| ≥ 11 hospital days in 3 years | 36 | 2,4 | 0,69 | high |
| F^33^ | Medicaid | C \| Top 10% cost-distribution | 12 | 3,3 | 0,75 | high |
| G^33^ | Medicaid | U \| ≥ 2 ED visits | 12 | 2,7 | 0,70 | high |
| H^61^ | Medicaid | C \| Top 1% cost-distribution | 12 | 4,7 | 0,72 | high |
| I^38^ | Hospitalized | C \| Top 10% for three consecutive years | 24 | 2,3 | 0,80 | unclear |
| J^62^ | General population | C \| Top 10% cost-distribution | 1 | 3,8 | 0,82 | high |
| K^30^ | General population | C \| Top 5% cost-distribution | 60 | 1,4 | 0,82 | unclear |
| L^63^ | Medicaid | C \| Top 5% cost-distribution | 12 | 7,9 | 0,82 | unclear |
| M^31^ | Veterans Affairs healthcare service | C \| Top 2% cost-distribution | 12 | 6,9 | 0,84 | low |
| N^34^ | General population | C \| Top 5% cost-distribution | 12 | 7,8 | 0,84 | unclear |
| O^35^ | General population | C \| Top 5% cost-distribution | 12 | 4,3 | 0,84 | low |
| P^64^ | General population | C \| Top 10% cost-distribution | 12 | 4,4 | 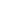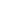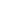0,84 | unclear |
| Q^65^ | General population | C \| Total costs | 12 | 9,6 | 0,85 | unclear |
| R^34^ | General population | U \| Hospital stay of ≥ 11 days | 12 | 6,7 | 0,85 | unclear |
| S^66^ | General population | C \| High-Cost trajectory | 12 | 6,5 | 0,86 | unclear |
| T^53^ | General population | C \| Top 5% cost-distribution | 12 | 8,6 | 0,87 | low |
| U^67^ | Medicare | C \| 2-Year High-Cost trajectory | 12 | 8,4 | 0,90 | high |
